# How have breeders adapted rice flowering to the growing region?

**DOI:** 10.1111/jipb.13785

**Published:** 2024-10-25

**Authors:** Asako Kobayashi, Mao Suganami, Hideki Yoshida, Yoichi Morinaka, Syuto Watanabe, Yoshie Machida, Genki Chaya, Fumihiro Nakaoka, Nobuhito Sato, Kotaro Miura, Makoto Matsuoka

**Affiliations:** ^1^ Rice Breeding Group Fukui Agricultural Experiment Station Fukui 918‐8215 Japan; ^2^ Faculty of Food and Agricultural Sciences, Institute of Fermentation Sciences Fukushima University Fukushima 960‐1296 Japan; ^3^ Faculty of Bioscience and Biotechnology Fukui Prefectural University Fukui 910‐1195 Japan

**Keywords:** breeding, flowering, GWAS, heading date, partial correlation analysis, rice

## Abstract

Flowering time is a crucial rice trait that influences its adaptation to various environments, cropping schedules, and agronomic characteristics. Rice breeders have exploited spontaneous mutations in heading date genes to regulate the flowering time. In the present study, we investigated how breeders in Fukui Prefecture regulated days to heading while developing promising rice varieties. Genome‐wide association studies (GWAS) identified *Hd1, Hd16*, and *Hd18* as the major genes controlling days to heading in the population. However, we suspected that this highly bred population might exhibit genomic stratification, which could lead to spurious or false correlations in the GWAS. Thus, we also conducted correlation and partial correlation analyses, which uncovered another key heading date gene, *Hd17*, that GWAS failed to detect because of its linkage disequilibrium with the major effect gene *Hd16*. Examination of haplotype frequencies across different breeding periods revealed that the early‐heading *Hd16* (*Hd16(E)*) and late‐heading *Hd17* (*Hd17(L)*) were increasingly co‐selected in the *Hd1* functional population. Varieties carrying this *Hd16(E)/Hd17(L)* combination exhibited days to heading in the range of 70–80, which corresponds to the peak temperature and sunshine period and is also optimal for grain quality and yield components in the Fukui environment. The present study highlights that it is imperative to remain vigilant for Type I (false positives) and Type II (false negatives) errors when performing GWAS on highly bred populations and to implement appropriate countermeasures by accounting for gene‐by‐gene interactions established through the breeding process. We also discuss the effectiveness of *Hd16(E)*, which is not used outside Japan for subtle days to heading control but is widely used in Japan at certain latitudes.

## Graphical abstract

Rice breeders use their experience and intuition to develop varieties with the optimum heading date for different growing areas; genome‐wide association studies and partial correlation analysis showed that Japanese breeders have achieved the desired heading date by cleverly combining four specific *Heading date* (*Hd*) genes, *Hd1*, *Hd16*, *Hd17*, and *Hd18*.

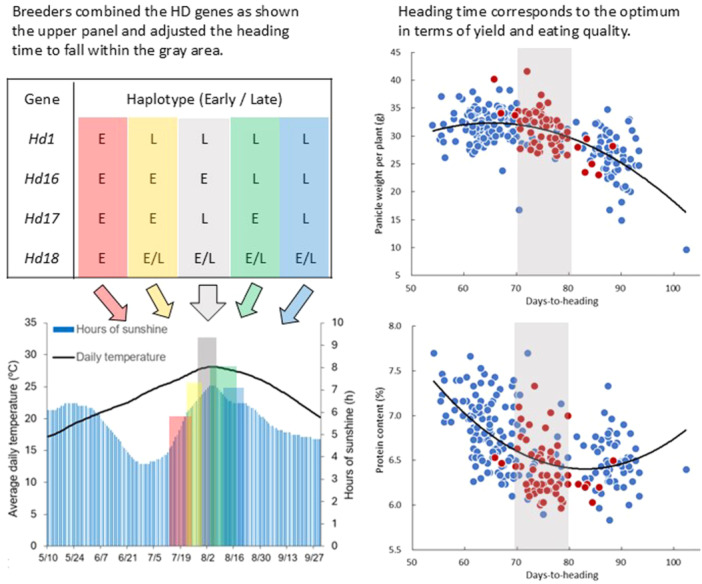

## INTRODUCTION

Flowering time is an important trait in adapting rice to different growing regions and cropping seasons. It is also important, even within a single location, as it enables the combination of different early‐to late‐heading varieties for a diversified cropping schedule and an optimized management plan that avoids the concentration of farming activities. Flowering time also influences various agronomic traits such as grain quality, plant height, culm stiffness, lodging degree, panicle length, disease resistance, and yield potential (Takeuchi et al., 2008; Hori et al., 2012, 2016). Therefore, rice breeders have regulated the flowering time in various growing areas using spontaneous mutations of flowering time regulatory genes (heading date (HD) genes), and molecular genetics has studied the molecular mechanism of flowering time using such spontaneous mutations, resulting in the identification of multiple quantitative trait loci (QTLs) and HD genes. To date, more than a dozen HD genes have been isolated and their biological functions have been elucidated (reviewed by Zhou et al., 2021; Vicentini et al., 2023). *Hd1* is one of the major HD genes in rice that directly regulates the expression of *EARLY HEADING DATE 1* (*Ehd1*), which is a direct inducer of the florigen genes, *Hd3a* and *RFT1*. *Ghd7/Hd4* and *DTH8/Hd5/Ghd8* repress heading under long‐day conditions by interacting with *Hd1* to repress *Ehd1* and florigen genes. *Hd2*/*OsPRR37*/*DTH7*/*Ghd7.1* encodes another transcriptional regulator that counteracts *Hd1*. These four genes, *Hd1*, *Ghd7*, *DTH8*, and *Hd2* are considered to be the core photoperiod genes for rice flowering time regulation and their combinations were also recently shown to significantly affect yield‐related traits (Sun et al., 2023). *Hd16* encodes a casein kinase I protein that phosphorylates Ghd7 and Hd2 to regulate their heading suppressor activity and acts as an enhancer of *Hd1* (Hori et al., 2013; Kwon et al., 2015; Nemoto et al., 2018). *Hd17* encodes an ELF3‐like protein that functions in the phytochrome pathway to regulate the expression of the HD and circadian genes to promote heading under long‐day (Yang et al., 2013; Saito et al., 2012; Itoh et al., 2019). The extensive interaction of these genes collaborating with other HD genes results in a sophisticated regulatory network that allows rice to adapt to different latitudes and growing seasons by precisely controlling flowering time.

Cross‐breeding of rice began in Japan in 1904 (Yamamoto, 1992), and thousands of varieties have been developed since then (e.g., Rice Variety database, NARO, 2023). Today, with these biological resources and the molecular genetic knowledge of the HD genes, it is possible to design the rice variety with the genotypes of the genes to suit the environment by using marker‐assisted selection. However, breeders have relied on their “experience and intuition” to select the flowering time that suited their growing area from a large number of rice plants. There are few examples of the successive use of such genetic resources and knowledge to breed varieties suitable for the growing environment. A representative example of such exceptional efforts is the control of flowering time in rice cultivated in Hokkaido in Japan and in Heilongjiang Province in China, which are located above 40°N, the northern limit of rice cultivation, where it was shown that mutations in *Ghd7* and *Hd2* are important to achieve extremely early heading (Li et al., 2015, 2018; Fujino et al., 2017, 2019; Chen et al., 2023).

Fukui Prefecture is located in the Hokuriku region, almost in the center of Japan (36°N, 136°E). It faces the Sea of Japan, which provides warm temperatures, moderate rainfall, and long hours of sunshine in summer, while the early arrival of autumn and moderate night temperatures during rice ripening makes the region suitable for rice production (Takami, 1995). Taking advantage of these regional characteristics, the Fukui Agricultural Experiment Station (FAES) started a rice breeding program in 1947, and in the course of approximately 80 years of rice breeding, more than 10 million individual rice plants have been tested, and 288 lines, with the number Etsunan (ETN), have been selected as promising lines to date (Table 1). “Etsunan” is the old name for the region where Fukui is located, and lines bred at FAES are given the name “Etsunan” plus a serial number. Only 37 lines are recognized as having superior traits different from those of previously released varieties and have been recognized as new varieties and given a special name and approval for cultivation. These include “Koshihikari” (ETN017) (Kobayashi et al., 2018), “Hanaechizen” (ETN146) (Horiuchi et al., 1992) and “Akisakari” (ETN208) (Tomita et al., 2009), which are widely grown throughout Japan as representative Japanese varieties.

**Table 1:** Breeding periods in Fukui Agricultural Experiment Station

Period	I	Ⅱ	Ⅲ	Ⅳ
Year	1949–1969	1970–1992	1993–2005	2006–2019
Main breeding targets	High yield and disease resistance	High eating quality	High eating quality	Tolerance to heat‐induced quality decline
Selection	Performance test and tolerance to diseases	→	→	→
		Sensory test for eating quality	→	→
			Amylose content of endosperm	
				Grain quality at high‐temperature ripening
Number of ETN lines	93	55	54	50
Early‐heading lines	38	33	20	23
Medium to late‐heading lines	55	22	34	27

The aim of this study was to elucidate how breeders regulated the “days to heading (DTH)” in an attempt to further expand and diversify the cultivation system with ETN lines bred over the past 70 years while adapting them to the environment of Fukui. We used two different statistical methods, a genome‐wide association study (GWAS) and a partial correlation analysis, to identify HD genes. The results showed that *Hd1*, *Hd16*, *Hd17*, and *Hd18* were the leading genes controlling DTH in the ETN population and that an artificially created linkage disequilibrium (LD) relationship was established between these genes in the breeding process. It was expected that it is often possible to create the desired trait by combining multiple genes in the breeding process; however, this leads to Type I and Type II errors in statistical analysis and erroneous conclusions. In this paper, using whole‐genome information and focusing on DTH, we investigated the genetic mechanisms underlying the development of a highly adapted population in a specific region through a 70‐year breeding process. To our knowledge, this comprehensive analysis of long‐term breeding effects on population adaptation has not been done before. In addition, we proposed specific strategies to address the challenges associated with conducting GWAS on such highly adapted populations in specific environments. These strategies take into account gene‐by‐gene (GxG) interactions and provide solutions to potential caveats in the analysis.

## RESULTS

### GWAS on the DTH of ETN lines

**Figure 1:**
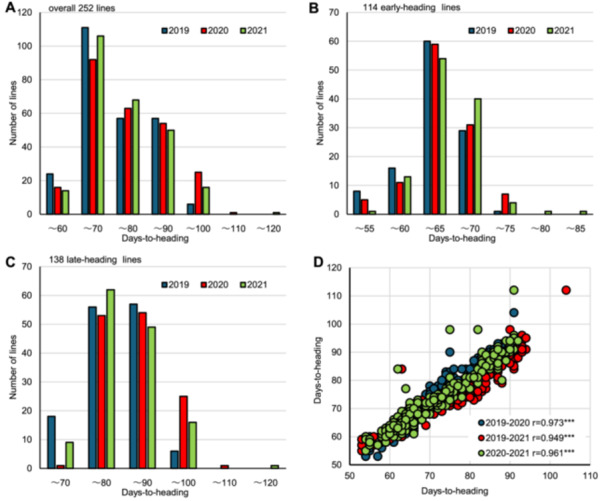
Days to heading (DTH) of ETN lines in 2019, 2020, and 2021 **(A**–**C)** Histogram of DTH of all 252 ETN lines, 114 early‐heading lines, and 138 late‐heading lines, respectively. **(D)** Correlation of DTH between years. Pearson's correlation coefficients (r) between 2 years are shown. *** shows adjusted *P*‐values by Holm's method <0.001.

The whole genomes of all 252 ETN lines bred between 1949 and 2019 were analyzed using next‐generation sequencing (NGS) and used as the ETN population panel (see Methods for details). These lines were classified as early‐heading (114 lines) or late‐heading (138 lines; sum of medium and late‐heading lines) according to their heading date at the time of establishment (Breeding Report of New Paddy Rice Lines by FAES, 1950–2020, Table S1). First, we examined the DTH of the ETN lines and found that they were distributed in the range of 54–92 d in 2019, 53–104 d in 2020 and 55–112 d in 2021 (Figure 1A), with high reproducibility throughout the 3 years (Figure 1D), confirming that genetic factors are highly involved in DTH. When DTH was examined for the early‐heading and late‐heading populations, it was found to be distributed between 53 and 84 d over the 3 years in the early‐heading lines (Figure 1B) and between 65 and 112 d in the late‐heading lines (Figure 1C), which was generally consistent with the classification at breeding.

Prior to the GWAS, we conducted principal component analysis (PCA) to examine the population structure based on the genomic structure of the overall, early, and late‐heading populations and found that all three populations were divided into two subpopulations according to PC1 (Figure S1). Therefore, we examined the correlation between PC1/PC2 scores and DTH to test whether this subpopulation structure could affect the GWAS and found no significant correlation in these three populations (Figure S2). Therefore, this subpopulation structure was not expected to have a significant effect on the GWAS for DTH.

**Figure 2:**
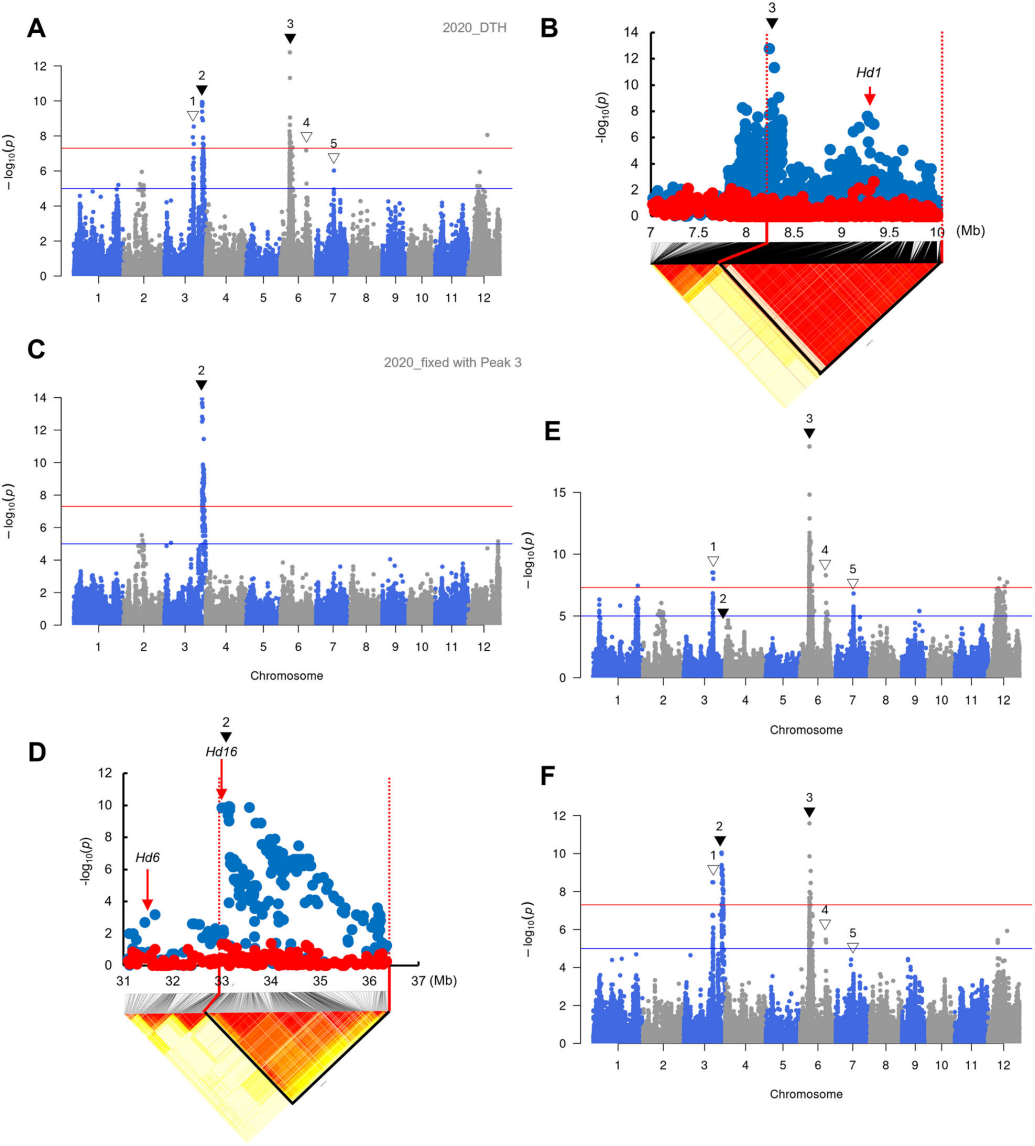
Genome‐wide association study (GWAS) for days to heading (DTH) in 252 overall ETN population in 2020 **(A)** Manhattan plot of GWAS for DTH. Genome‐wide thresholds were set to the significance threshold (*P* = 5.0 × 10^−8^; red) and suggestive threshold (*P* = 1.0 × 10^−5^; blue). Arrowheads indicate the peaks that are above the threshold for all 3 years 2019, 2020, and 2021. Black arrowheads indicate the peaks corresponding to known heading date (HD) genes and white arrowheads indicate the peaks considered to be false positives. **(B**, **D)** Local Manhattan plot and LD heat map surrounding **(B)** Peak 3 (7–10 Mb on Chr. 6) and **(D)** Peak 2 (31–37 Mb on Chr. 3). The red arrow indicates the position of HD genes, *Hd1* **(B)**, *Hd6* and *Hd16* **(D)**. Plots show the results of GWAS performed without (blue) or with (red) the polymorphism with the highest −log_10_(*P*) within the peak region. **(C**, **E**, **F)** Manhattan plots of GWAS using the polymorphism with the highest −log_10_(*P*) within Peak 3 **(C)** and Peak 2 **(E)** and using the causative polymorphism of *Hd6* **(F)**.

Figure 2A shows the GWAS results using the 2020 DTH data, whereas Figure S3A, E show the results for 2019 and 2021, respectively. There were two peaks on chromosome (Chr) 3 (Peaks 1 and 2), two peaks on Chr 6 (Peaks 3 and 4) and one peak on Chr 7 (Peak 5). The reliability of these results was confirmed by quantile–quantile (Q–Q) plotting. No problematic inflation was found, suggesting no statistical problems (Figure S4).

First, we focused on Peak 3 of Chr 6 with the highest −log_10_(*P*) (Figure 2A). The LD of this peak region contained the *Hd1* gene, but there were two distinct peaks (Figure 2B). Yano et al. (2016) reported that in the Japanese rice population, *Hd1* often has multiple haplotypes with functional mutations (Figure S5A), resulting in the formation of peaks outside the open reading frame (ORF) region of the *Hd1* gene itself. As it was considered that this applies to Peak 3, we re‐performed the GWAS including the polymorphism with the highest −log_10_(*P*) within the Peak 3 region (hereafter referred to as “peak highest polymorphism”) as a fixed effect and found that Peak 3, including a polymorphism that alters the function of *Hd1* (red dots in Figure 2B), completely disappeared (Figure 2C), confirming the above prediction that Peak 3 is caused by polymorphism of *Hd1*. Furthermore, Peaks 1, 4, and 5 disappeared, leaving only Peak 2, when including the fixed effect of Peak 3 in this GWAS (Figure 2C). Thus, these peaks were predicted to be false positives (Type I errors) caused by their LD relationship with *Hd1*. Similar results were obtained for the 2019 and 2021 DTH datasets (Figure S3B, F).

There were two HD genes, *Hd16* (Hori et al., 2013) and *Hd6* (Takahashi et al., 2001), in the Peak 2 region (Figure 2D). The causal polymorphism affecting *Hd16* function (33,002,789; Figure S5B) had −log_10_(*P*) = 9.85, whereas that of *Hd6* (31,512,460; Figure S5C) had −log_10_(*P*) = 0.85. Furthermore, when the GWAS was performed using the highest polymorphism of Peak 2 (33,147,698) as a fixed effect, Peak 2, including the causal polymorphism of *Hd16*, almost completely disappeared (Figure 2E; red dots in Figure 2D). However, when the causal polymorphism of *Hd6* was used as a fixed effect, Peak 2 was almost unaffected (Figure 2F; gray dots in Figure S6). Similar results were obtained for the 2019 and 2021 DTH data (Figure S3C, D, G, H), indicating that the gene responsible for Peak 2 was *Hd16*. These results indicated that DTH is exclusively regulated by *Hd1* and *Hd16* in the ETN population.

**Figure 3:**
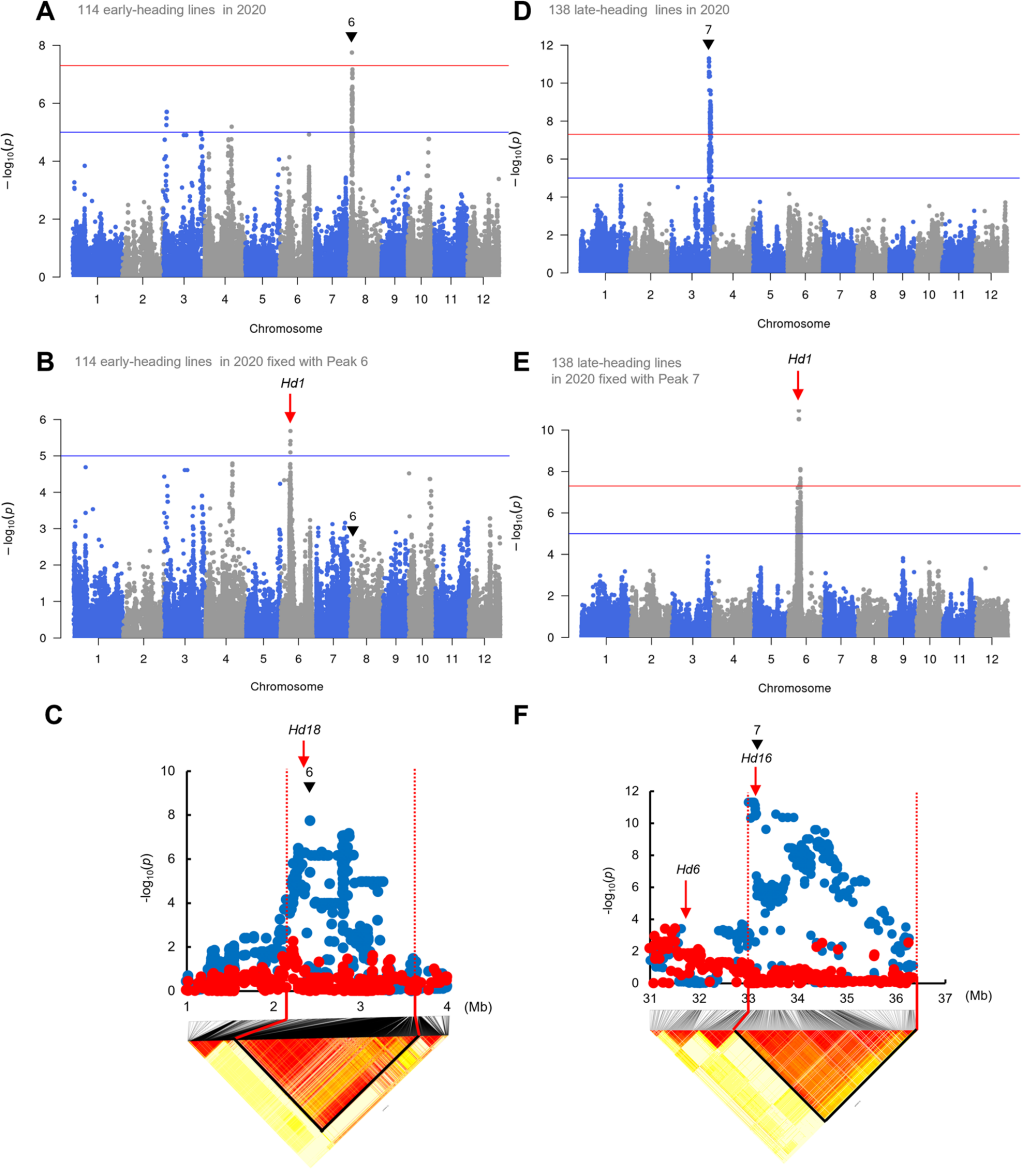
Genome‐wide association study (GWAS) for days to heading (DTH) in 114 early‐heading and 138 late‐heading lines **(A**, **D)** Manhattan plots of GWAS for DTH in 114 early‐heading **(A)** and 138 late‐heading lines **(D)** in 2020. Genome‐wide thresholds were set to the significance threshold (*P* = 5.0 × 10^−8^; red) and suggestive threshold (*P* = 1.0 × 10^−5^; blue). Black arrowheads indicate the peaks that are above the threshold for all 3 years 2019, 2020, and 2021. **(B**, **E)** Manhattan plots of GWAS using the polymorphism with the highest −log_10_(*P*) within Peak 6 **(B)** and Peak 7 **(E)**. **(C**, **F)** Local Manhattan plot and LD heat map surrounding the peak regions **(C)** Peak 6 (1–4 Mb on Chr. 8) and **(F)** Peak 7 (31–37 Mb on Chr. 3). The red arrow indicates the position of heading date (HD) genes, *Hd18* **(C)**, *Hd6* and *Hd16* **(F)**. Plots show the results of GWAS performed without (blue) or with (red) the polymorphism with the highest −log_10_(*P*) within the peak region.

We then performed GWAS on two subpopulations: early‐heading and late‐heading populations (Figure 3). In the early‐heading population, only one peak, Peak 6 on Chr 8, exceeded the threshold throughout the 3 years (Figures 3A, S7A, B). This peak region contains *Hd18* (Shibaya et al., 2016), and a GWAS with the highest polymorphism of Peak 6 as a fixed effect resulted in the complete disappearance of Peak 6, including the causal polymorphism of *Hd18* (2,388,554; Figure S5D) (Figures 3B, S7C, D; red dots in Figure 3C). The only peak that exceeded the threshold throughout the 3 years in the late‐heading population was Peak 7 on Chr 3 (Figures 3D, S7E, F). This coincided with the position of Peak 2 in the overall GWAS (Figure 2A); therefore, the same analysis confirmed that *Hd16* was responsible for Peak 7 (Figures 3E, F, S7C–H). The GWAS of the early‐heading and late‐heading populations showed that no significant peak was detected in the *Hd1* region, which showed the largest effect in the overall population, suggesting that *Hd1* discriminates “early and late” of the DTH of ETN lines. Indeed, 101 (89%) of the 114 early‐heading lines had non‐functional/early‐heading *Hd1* (Hap. C and Hap. D in Figure S5A), whereas 131 (95%) of the 138 late‐heading lines had functional/late‐heading *Hd1* (Hap. A and Hap. B) (Figure S8). In contrast, the *Hd1* peak appeared in the early and late populations when the highest peak polymorphisms of Peak 6 and Peak 7 polymorphisms were used as fixed effects (Figures 3B, E, S7C, D, G, H; red dots in Figure S9). This could be because the effects of the small number of functional (11%) and non‐functional (5%) *Hd1* in the early‐ and late‐heading populations, respectively, became apparent when the effects of *Hd16* and *Hd18* were excluded. Therefore, we re‐performed the GWAS after dividing the ETN population into *Hd1* non‐functional and functional groups. The results showed that the *Hd1* non‐functional and functional populations were similar to the GWAS of the early‐ and late‐heading populations (Figure S10A–F) in the *Hd1* non‐functional population, a new peak appeared at the long arm end of Chr 3 in addition to Peak 6/*Hd18*. This peak corresponded to the position of Peak 2 in the overall GWAS (Figures 2A, S3A, E) and Peak 7 in the late GWAS (Figures 2D, S7E, F), and the same analysis as above confirmed that the gene responsible for this peak was *Hd16* (Figure S10G–I). The combined GWAS results showed that the ETN population can be broadly divided into early‐heading and late‐heading groups according to *Hd1* and that *Hd18* and *Hd16* have a significant effect on DTH in the early‐heading group and *Hd16* in the late‐heading group. In further analyses, we distinguished between early‐ and late‐heading populations based on *Hd1* non‐functional and functional haplotypes instead of distinguishing between early and late populations based on the Breeding Report by FAES.

### Effect of polymorphisms in HD genes on DTH

The GWAS results showed that *Hd1*, *Hd16*, and *Hd18* were the three main regulators of DTH in ETN lines. However, considering the breeding history of the ETN population, we suspect that the ETN population may have formed links between loci across chromosomes. Therefore, we searched for DNA polymorphisms in the 22 HD genes reported to date (Table S2; see Methods for details) and identified *Hd2*, *Hd6*, and *Hd17*, whose polymorphisms were found in more than 5% of the lines in the ETN population, in addition to the three genes mentioned above (Figure S5C, E, F). Table 2 shows the reference and alternative haplotypes of the polymorphisms of these six HD genes, functional/non‐functional, and early/late heading, based on their known information and average DTH.

**Table 2:** SNPs or haplotypes of flowering genes within the ETN population

Gene	*Hd1*	*Hd2*	*Hd6*	*Hd16*	*Hd17*	*Hd18*
Function^a^	Short‐day promoter/Long‐day repressor	Circadian rhythm/Short‐day promoter/Long‐day repressor	Long‐day repressor	Long‐day repressor	Circadian rhythm/Long‐day promoter	Constitutive promoter
Chr.	6	7	3	3	6	8
SNP position	9,336,861 (Hap B); 9,337,236 (Hap C); 93,38,004 (Hap D)	2,96,23,803	3,15,12,460	3,30,02,789	22,35,191	23,88,554
Reference allele^b^	Hap A, Hap B	G	T	G	T	A
Functionality	F	F	N	F	F	HF
Effects	L	L	E	L	E	E
Mean The	78.8	72.8	71.6	76.2	74.9	72.5
Number of lines	144	224	224	125	88	162
Alternative allele	Hap C, Hap D	A	A	A	C	G
Functionality^c^	N	N	F	N	LF	F
Effect^d^	E	E	L	E	L	L
Mean DTH^e^	63.3	65.7	75.4	67.9	70.6	71.6
Number of lines	108	28	28	127	164	90

^a^ Role in rice flowering pathway. Referenced from Lee and An (2015), and Li et al. (2018).

^b^ Reference alleles are basically the Nipponbare alleles.

^c^ Chr., chromosome; F, functional; HF, high‐functional; LF, low‐functional; N, non‐functional.

^d^ Effect on the days to heading (DTH) estimated from physiological functions; E: early heading (−DTH), L: late heading (+DTH).

^e^ Mean of days to heading of lines harboring the corresponding allele.

We then performed a correlation analysis between these genes and DTH, as well as a partial correlation analysis, considering false correlations caused by LD relationships between these HD genes (3, 4, 5). Partial correlation analysis is useful not only for detecting false correlations but also for revealing false negative correlations. We used the point‐biserial correlation coefficient, a measure of the correlation between a binary variable and a continuous variable, for the correlation between genotypes and DTH and the phi coefficient, a measure of the association between two binary variables, for the correlation between HD genes. We coded haplotypes with lower activity as 0 and those with higher activity as 1 for each gene function. Thus, genes promoting early heading were negatively correlated with DTH because higher functional haplotypes decreased DTH. Conversely, genes suppressing heading were positively correlated with DTH because higher functional haplotypes increased DTH. In the correlation analysis, *Hd1* showed a significantly higher positive correlation with DTH throughout the 3 years (Table 3). *Hd16* also showed a significant positive correlation over the 3 years, whereas *Hd2* showed a significant but weak positive correlation. In 2019 and 2021, *Hd17* also showed a significantly weak positive correlation. In contrast, the partial correlation analysis showed that the correlations between *Hd2*, *Hd17* and DTH disappeared, whereas the correlation with *Hd16* increased. The reason for the disappearance of the correlation between *Hd2* and DTH could be due to a false correlation (Type I error) resulting from the effect of *Hd1* on *Hd2*, as the LD relationship between *Hd2* and *Hd1* was observed (Table S3, phi coefficient = 0.30). The weakly positive correlation of *Hd17* observed in the correlation analysis was negatively correlated in the partial correlation analysis, which is consistent with the original function of *Hd17*, as discussed below.

**Table 3:** Point‐biserial correlations between heading genes and DTH (overall ETN population)

		Point‐biserial correlation			Partial point‐biserial correlation		
Gene	Effect on DTH	2019	2020	2021	2019	2020	2021
*Hd1*	+	0.75***	0.78***	0.71***	0.83***	0.86***	0.78***
*Hd2*	+	0.21*	0.23**	0.20*	0.08	0.14	0.08
*Hd6*	+	0.12	0.08	0.14	0.13	0.11	0.14
*Hd16*	+	0.42***	0.37***	0.43***	0.64***	0.65***	0.60***
*Hd17*	−	0.21*	0.16	0.22**	−0.07	−0.15	−0.03
*Hd18*	−	0.06	0.03	0.06	−0.13	−0.23**	−0.10

*n* = 252 in 2019 and 2021, *n* = 248 in 2020.

Effect on the days to heading (DTH) estimated from physiological functions; −(−DTH); early heading, +(+DTH); late heading.

*^,^ **^,^ *** shows adjusted *P*‐values by Holm's method <0.05, <0.01, <0.001, respectively.

**Table 4:** Point‐biserial correlations between heading genes and DTH (*Hd1* non‐functional population)

		Point‐biserial correlation			Partial point‐biserial correlation		
Gene	Effect on DTH	2019	2020	2021	2019	2020	2021
*Hd2*	+	−0.04	0.09	−0.04	0.07	0.24	0.05
*Hd6*	+	0.26	0.10	0.23	0.12	0.01	0.10
*Hd16*	+	0.53***	0.58***	0.46***	0.47***	0.57***	0.39***
*Hd17*	−	0.05	−0.08	0.06	−0.07	−0.16	−0.05
*Hd18*	−	−0.38***	−0.43***	−0.36**	−0.33**	−0.46***	−0.31*

*n* = 108 in 2019 and 2021, *n* = 107 in 2020.

Effect on the days to heading (DTH) estimated from physiological functions; −(−DTH); early heading, +(+DTH); late heading.

*^,^ **^,^ *** shows adjusted *P*‐values by Holm's method <0.05, <0.01, <0.001, respectively.

**Table 5:** Point‐biserial correlations between heading genes and DTH (*Hd1* functional population)

		Point‐biserial correlation			Partial point‐biserial correlation		
Gene	Effect on DTH	2019	2020	2021	2019	2020	2021
*Hd2*	+	−0.05	−0.10	−0.04	0.19	0.10	0.18
*Hd6*	+	0.32**	0.31**	0.33***	0.22	0.21	0.24
*Hd16*	+	0.76***	0.70***	0.73***	0.77***	0.72***	0.73***
*Hd17*	−	0.20	0.15	0.22	−0.26*	−0.29**	−0.20
*Hd18*	−	0.03	−0.01	0.05	−0.23	−0.26*	−0.18

*n* = 144 in 2019 and 2021, *n* = 141 in 2020.

Effect on the days to heading (DTH) estimated from physiological functions; −(−DTH); early heading, +(+DTH); late heading.

*^,^ **^,^ *** shows adjusted *P*‐values by Holm's method <0.05, <0.01, <0.001, respectively.

We performed a similar analysis for *Hd1* non‐functional and functional populations. In the *Hd1* non‐functional population, *Hd16* and *Hd18* were significantly correlated, which was consistent with the GWAS results (Table 4). Regarding the correlations between genes, *Hd17* and *Hd6* showed a high correlation (Table S4), but neither showed a significant correlation with DTH, suggesting that they are not involved in DTH regulation. In the *Hd1*‐functional population, a strong correlation was found for *Hd16*, which was consistent with the GWAS results, whereas *Hd6* showed a moderate correlation over the 3 years (Table 5). The moderate correlation of *Hd6* was assumed to be due to a false correlation (Type I error) due to *Hd16*, as the partial correlation analysis showed that the correlation was no longer significant, and its LD relationship with *Hd16* was observed (Table S5, phi coefficient = 0.29). In contrast, *Hd17* showed a positive, although not significant, relationship with DTH in the correlation analysis, whereas the partial correlation analysis showed a negative relationship (Table 5). We suspected the results of the correlation analysis because, according to its known function, this gene should promote heading date and, therefore, should show a negative correlation with DTH (Table 2). In fact, the LD relationship between *Hd17* and *Hd16* was observed (phi coefficient = 0.42, Table S5), and the positive association in the correlation analysis could be due to a Type II error, where the effect of *Hd17* was offset by *Hd16* (see Discussion).

### Transition of the HD gene haplotypes in the *Hd1* functional population

**Figure 4:**
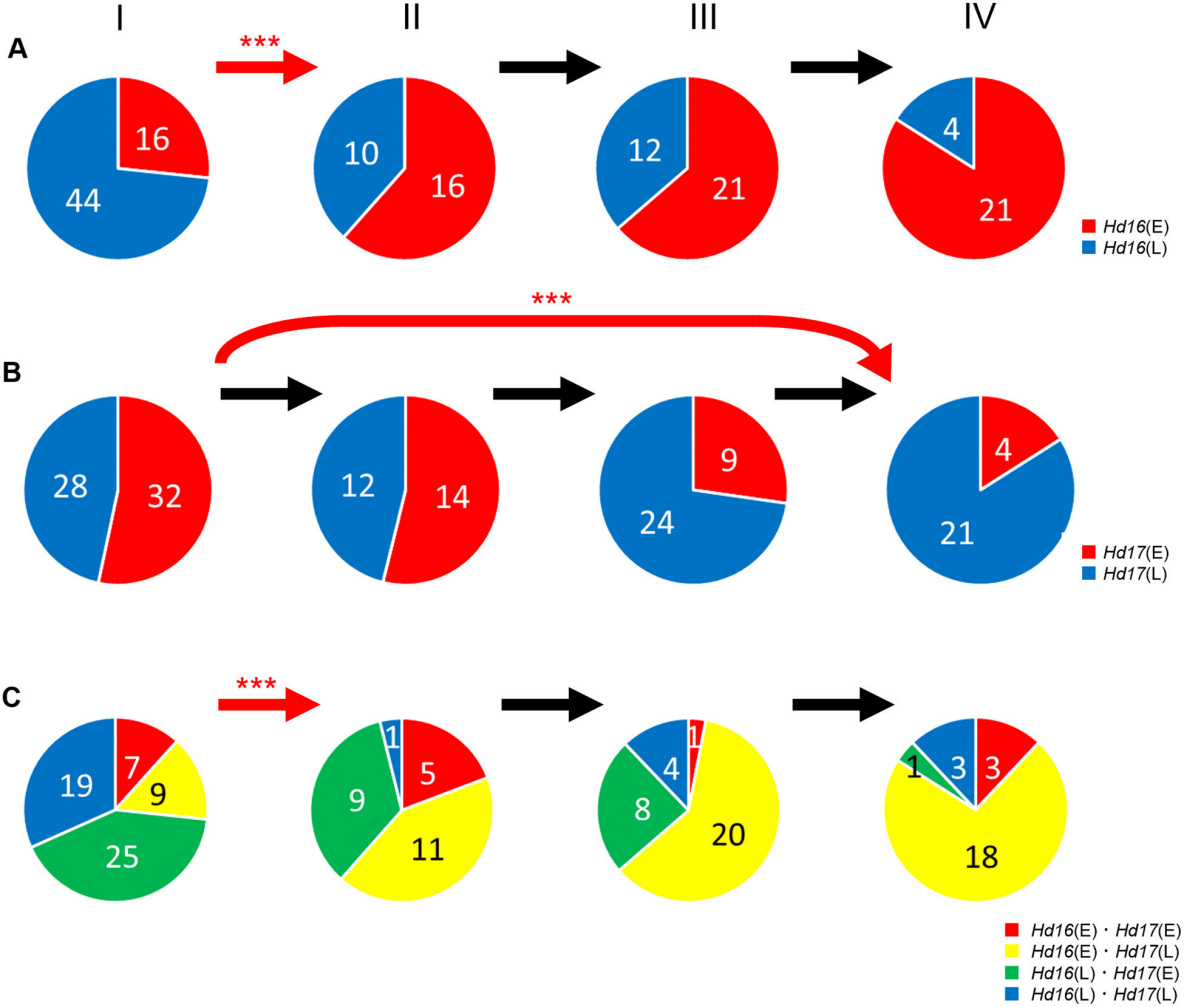
**Transition of haplotypes of** ***Hd16*** **and** ***Hd17*** **in** ***Hd1*** **functional population during the breeding process in Fukui** **(A**–**C)** Haplotype ratios of *Hd16* **(A)** and *Hd17* **(B)**, and the ratio of “*Hd16*(E) and *Hd17*(L)” combination during the breeding process, Periods I–IV (Table 1). “E” means earlier heading haplotype, “L” means later heading haplotype. The number inside the circle represents the number of lines. *** The above arrow indicates the significance at the 0.1% level, in a Fisher's exact test that allows rejection of the hypothesis that the ratio of haplotypes or polymorphisms in one period was maintained in the next period.

In the above analysis, we found the LD relationship between *Hd16* and *Hd17* in the *Hd1* functional population (Table S5). We suspected that this occurred during the breeding process and compared the haplotype ratios for *Hd16* and *Hd17* for each breeding period (Figure 4). The results showed that the frequency of early‐heading *Hd16* (*Hd16(E)*) increased significantly from Periods I to II, remained constant from Periods II to III, increased again from Periods III to IV, and finally accounted for 84% (Figure 4A). In contrast, the late‐heading *Hd17*, *Hd17(L)*, did not change from Periods I to II, increased from Periods II to III, and further increased from Periods III to IV, thus being significantly selected when comparing Periods I and IV (Figure 4B). We also calculated the Nei's genetic distance between Periods I and IV and confirmed significant peaks for *Hd16* and *Hd17* (Figure S11). The transition of the “*Hd16(E)* and *Hd17(L)*” set showed a significant increase from Periods I to II, followed by a continuing increase, and finally 72% of lines in Period IV (Figure 4C).

### Why had the combination of “*Hd16(E)* and *Hd17(L)*” been selected in the functional *Hd1* population?

**Figure 5:**
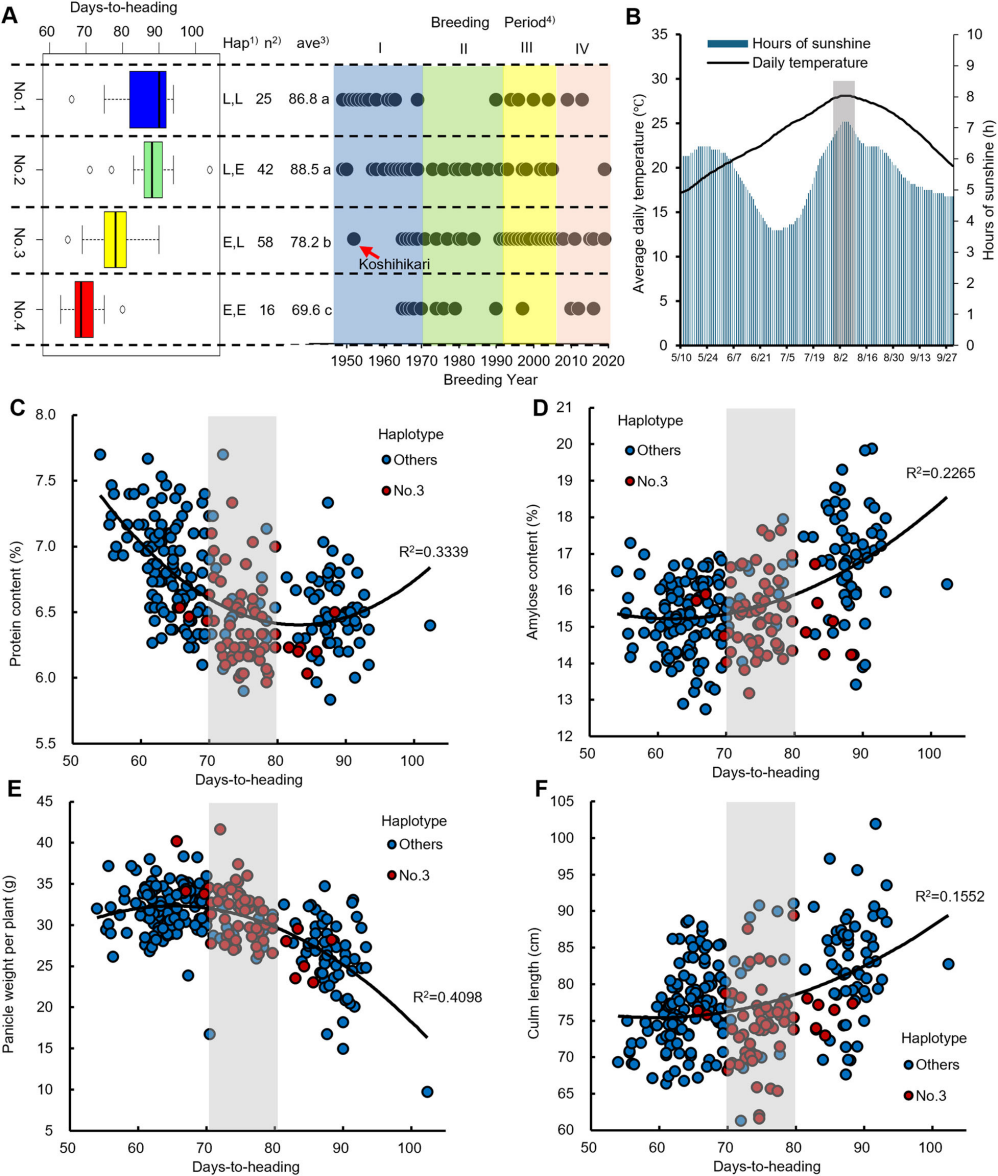
**Variation in days to heading (DTH) in** ***Hd1*** **functional population by combinations of** ***Hd16*** **and** ***Hd17*** **haplotypes** **(A)** Boxplot of DTH for each group classified by haplotype combination of heading date (HD) genes, and the breeding year of the ETN lines belonging to each group. (1) Haplotype of *Hd16* and *Hd17*, L: Later heading haplotype, E: Earlier heading haplotype. (2) Number of lines. (3) Means denoted by a different letter indicate significant differences at *P*‐value < 0.05 by Tukey–Kramer multiple comparison test. (4) See Table 1. The gray area of days to heading shows the average ± *SD* of DTH in 2020 induced by the haplotype No. 3. **(B)** Average daily temperature and hours of sunshine for the 30‐year period from 1991 to 2010. **(C**–**F)** Relationships between DTH and protein content in brown rice **(C)**, amylose content in polished rice **(D)**, panicle weight per plant **(E)**, and culm length **(F)**. The gray area of DTH shows the average ± *SD* of the average DTH for 2019, 2020 and 2021, induced by haplotype No. 3. Red plots indicate haplotype No. 3 lines and blue plots indicate others.

We studied the relationship between DTH and the year of establishment of each ETN line divided into four groups based on the *Hd16* and *Hd17* haplotypes of the functional *Hd1* population (Figures 5A, S12A, B). The average DTH of the *Hd16(L)* group (Groups No. 1 and No. 2) was 87.9 d in 2020, whereas that of the *Hd16(E)* group (Groups No. 3 and No. 4) was 76.3 d, and the difference was significant at the 5% level throughout the 3 years. In contrast, the effect of *Hd17* on DTH was not significant in *Hd16(L)* for any of the 3 years (No. 1 vs. No. 2), but the difference in DTH was 6.2 d in 2019, 8.6 d in 2020 and 5.8 d in 2021 in the *Hd16(E)* group (No. 3 vs. No. 4), which were significant except in 2021. In this population, the largest group No. 3 (*Hd16(E)/Hd17(L)*) accounted for about 40% (58 lines), including “Koshihikari” (ETN017), most were established in Periods III and IV (Figure 5A), and their DTH (means ± *SD*) ranged from 73.3 to 83.0 d in 2020, and 70.7 to 80.3 d over the 3 years (Figure S12; gray area in Figure 5A). We also investigated the relationship between the 70–80 DTH of haplotype No. 3 and average temperature and sunshine, as well as rice grain quality and yield. The results showed that this DTH period coincided with peak temperature and sunshine (Figure 5B) and was also within the appropriate range for rice grain quality and yield (Figure 5C–F). That is, when the DTH was less than 70 d, the protein content of brown rice increased (Figure 5C), whereas when the DTH was 80 d or more, the amylose content of polished rice increased (Figure 5D) and the eating quality of rice should have decreased (Inatsu, 1988). In addition, panicle weight per plant decreased when DTH was 80 d or more (Figure 5E) and the risk of lodging increased with increasing stem length (Figure 5F). These results strongly suggest that a DTH of 70–80 is optimally adaptive for these traits as it coincides with the peak temperature and sunlight levels in the Fukui growing environment, which explains why the haplotype combination *Hd16(E)*/*Hd17(L)* was selected in the FAES breeding and relevance.

### Regulation of DTH in the *Hd1* non‐functional population

Because *Hd16* and *Hd18* were involved in DTH control in the *Hd1* non‐functional population, we compared DTH and the year of establishment for each of the four haplotype combinations (Figure S13A–C). When the effect of *Hd16* on DTH was compared in the same *Hd18* background (“No. 5 vs. No. 7” and “No. 6 vs. No. 8”), it was significant at the 5% level in all cases except for “No. 6 vs. No. 8” in 2021. In contrast, there was no significant difference in any of the 3 years when the effect of *Hd18* was compared for the same *Hd16(E)* background (“No. 7 vs. No. 8”), while a significant difference was observed for *Hd16 (L)* (“No. 5 vs. No. 6”) in 2020 and 2021. The association between these four haplotype groups and their years of establishment showed no evidence of selection for specific haplotype lines (Figure S13D–F). As shown in Figure 5, early‐heading lines with less than 70 DTH have the disadvantages of higher protein content and lower eating quality in Fukui's growing environment, but they also have the advantage of avoiding the concentration of harvesting operations and being the first to supply new rice to the market. Therefore, there is a demand for early‐heading varieties despite their lower eating quality. To meet this demand, breeders have made subtle adjustments to *Hd16* and *Hd18* within a short DTH range under non‐functional *Hd1* (see Discussion).

### Breeding use of the early‐heading *Hd16(E)* haplotype

**Figure 6:**
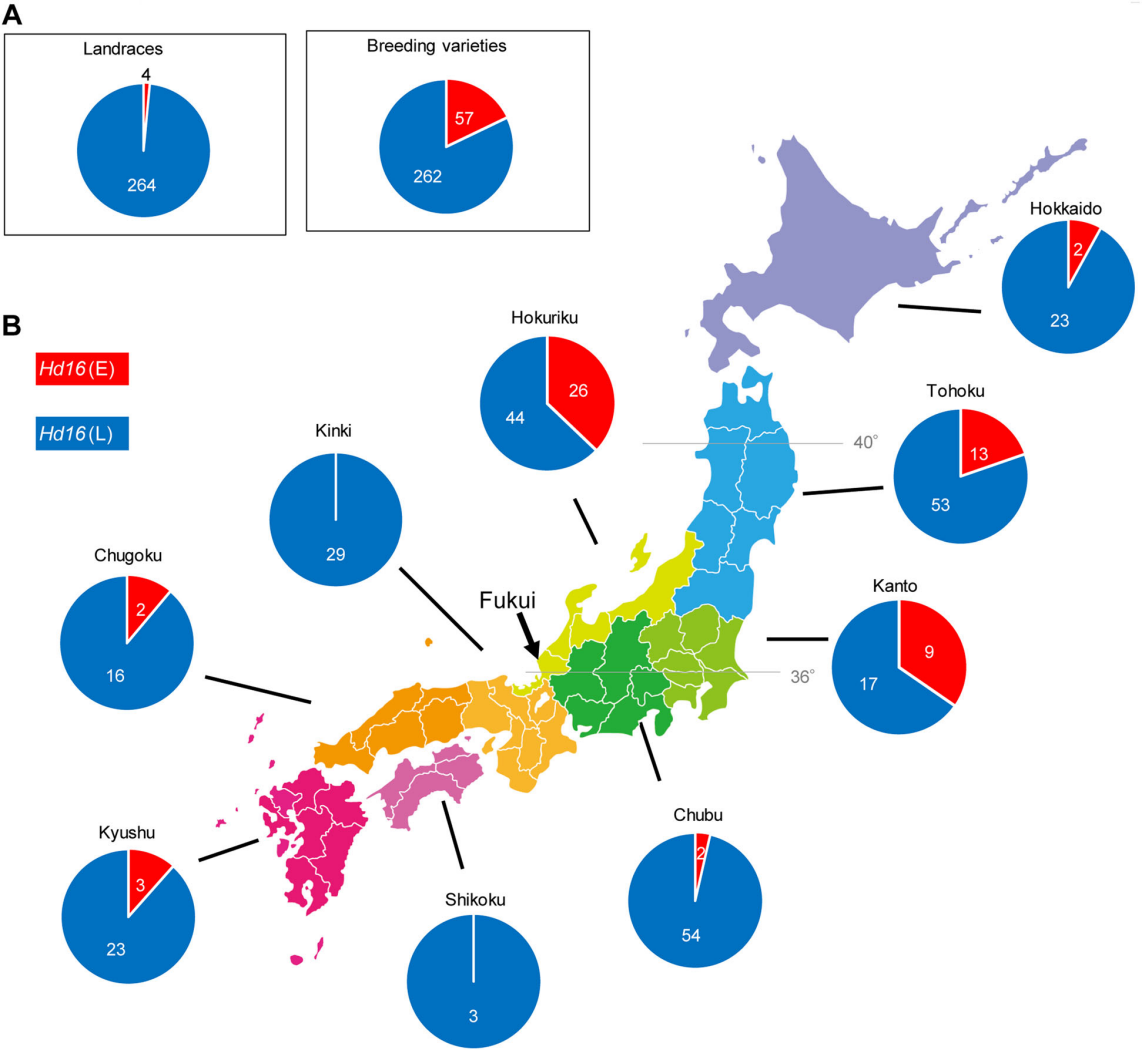
***Hd16*** **haplotype ratio in each region in Japan** **(A)** The pie charts in the box show the ratio of the earlier heading haplotype of *Hd16* and the later heading haplotype of *Hd16* in Japanese landrace and breeding varieties. **(B)** *Hd16* haplotype ratio on the map of Japan shows the ratio of *Hd16* alleles for each administrative region category for the breeding varieties.

These results indicated that *Hd16* is important in DTH in both *Hd1* non‐functional and functional populations. As *Hd16* was originally discovered as the gene responsible for the QTL modifying DTH in “Koshihikari” (ETN017) and “Nipponbare” (Hori et al., 2013), it makes sense that *Hd16* was used as an important gene controlling DTH in the ETN panel, because the ETN population has been bred for over 30 years using “Koshihikari” as an excellent mother with high eating quality. We investigated whether the high frequency of use of *Hd16* in this ETN panel was acceptable in other areas of Japan and abroad. We examined the frequency of *Hd16(E)* in 268 landraces and 319 modern varieties in Japan and found that only four (1.5%) of the landraces had this derived haplotype (two of the four lines in Table S6 have the same name, “Moritawase,” but these have different genome structures), whereas 57 (17.9%) of the modern varieties have *Hd16(E)*, indicating that this haplotype has increased with the progress of breeding also outside of Fukui (Figure 6A). Interestingly, the distribution of *Hd16(E)* varieties was localized in the 36°–40°N region (Hokuriku, Kanto, and Tohoku regions), and its frequency decreased north and south of this latitude (Figure 6B). The four landraces with *Hd16(E)* were from Tohoku, suggesting that *Hd16(E)* has long been used as a haplotype for convenient DTH in this region. Hori et al. (2013) reported that *Hd16(E)* originated from a mutation that occurred in the landrace “Moritawase.” However, other landraces, “Kamenoo” and “Sekiyama,” both of which were recorded earlier than “Moritawase” (Nishio and Fujimaki, 2020), also have *Hd16(E)* (Table S6), indicating that this haplotype has been maintained in the northeastern region of Japan since ancient times. We also investigated its overseas distribution using the Rice Var Map (https://ricevarmap.ncpgr.cn/), which showed that 1.6% was present in temperate *japonica* and 1.1% in intermediate subpopulations, whereas it was absent in other subpopulations (Table S7). We then further investigated the *Hd16(E)* varieties in foreign countries using the IRRI 3K rice data (Wang et al., 2018), and found one landrace (“Heibiao,” China), as well as “TIMICH108” (Romania) and “DECHANGBYEO” (Korea), which were indistinguishable from landraces or modern varieties, suggesting that *Hd16(E)* may have originally existed outside Japan.

## DISCUSSION

### Limitations of GWAS and its countermeasures

**Figure 7:**
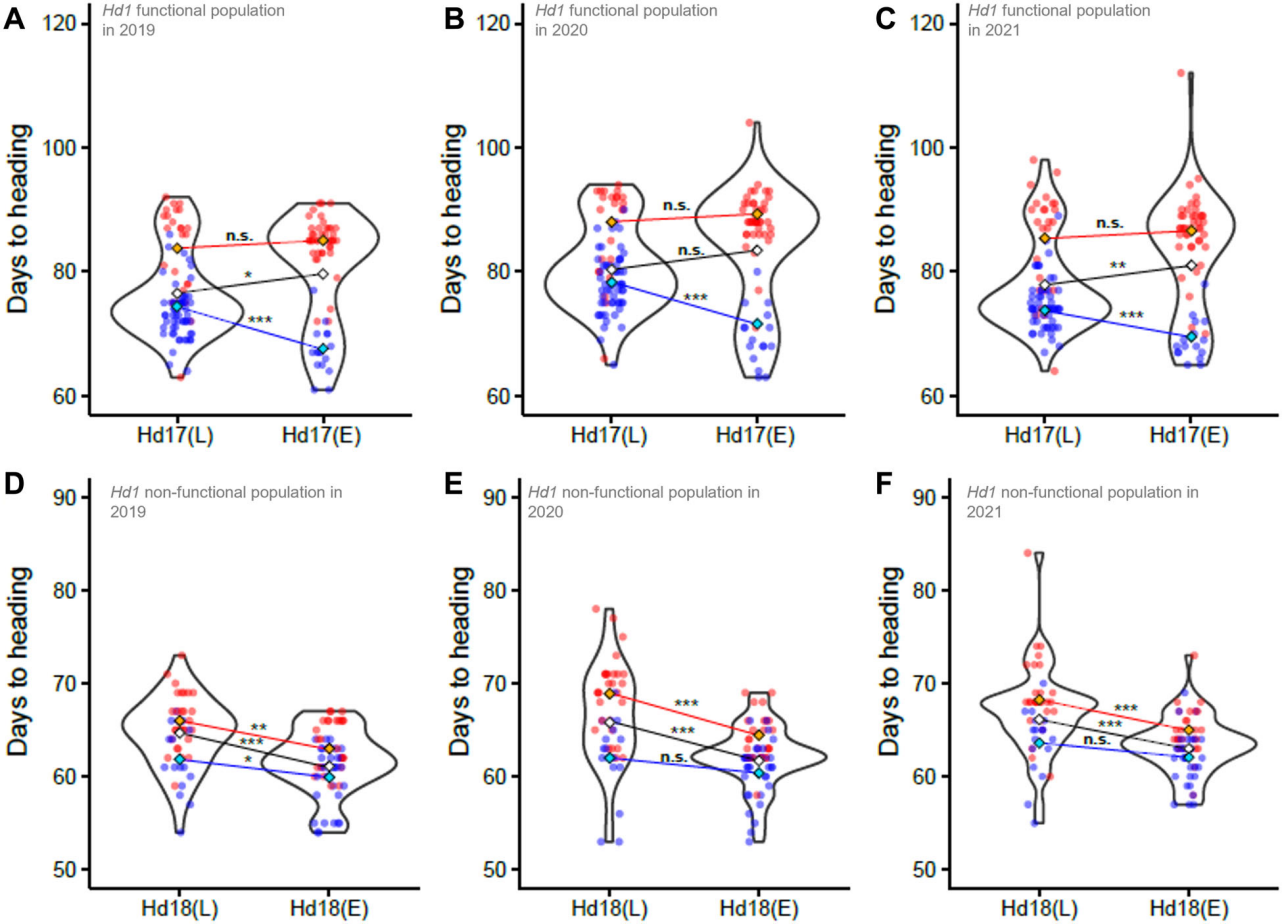
Gene‐by‐gene (G × G) interaction of heading date (HD) gene **(A**–**C)** Violin plots for days to heading (DTH) in *Hd1* functional population. **(D**–**F)** Violin plots for DTH in *Hd1* non‐functional population. Blue dots indicate lines with the *Hd16(E)* haplotype and red dots indicate lines with the *Hd16(L)* haplotype. The diamonds indicate the average of DTH in lines with the *Hd17(L)* haplotype and in lines with the *Hd17(E)* haplotypes, respectively. *, **, *** shows *P*‐values by two‐sided Student's *t*‐test <0.05, <0.01, <0.001, respectively. The straight line connecting these averages is the regression line.

It is well known that population stratification or structure can lead to spurious or pseudo‐correlations in GWAS (Richardson, 2017; Uffelmann et al., 2021). In this study, we used the ETN population, which may have structural stratification of the genome after years of repeated manipulation, to select good lines in certain environments; therefore, we suspected a risk of finding spurious or pseudo‐correlations in the GWAS. Indeed, when the peak highest polymorphism of the top peak, Peak 3, was used as a fixed effect in the GWAS of the overall ETN population, all peaks except Peak 2 disappeared (Figure 1), confirming that our hypotheses were correct. Therefore, we performed GWAS using the peak highest polymorphisms of the peak assumed to be true positives as a fixed effect to eliminate false‐positive peaks. However, although this method can eliminate false positives, it cannot identify loci because of false negatives. Therefore, we investigated the relationship between the causal polymorphisms of known HD genes and DTH using correlation and partial correlation analyses. We identified *Hd17*, which was not detected in the GWAS. The mechanism for the failure to detect *Hd17* in the GWAS was investigated using Figure 7A–C: in the *Hd1* functional population, *Hd17(L)* lines were dominant in the *Hd16(E)* group (blue dots), whereas *Hd17(E)* lines were dominant in the *Hd16(L)* group (red dots). Consequently, the average DTH of the *Hd17(L)* population (white diamonds) was shorter than that of the *Hd17(E)* population, leading to the opposite result of its intrinsic function. As *Hd16* and *Hd17* are located on different chromosomes, Chrs 3 and 6, respectively, the correlation between the two genes was not due to their physical neighborhood positions but was the result of artificial selection. Thus, by cleverly exploiting the antagonism between *Hd16(E)* and *Hd17(L)* (see below), the breeders succeeded in creating a GxG relationship that gave DTH suitable for the Fukui growing area (Figure 4C). The present study revealed that it is imperative to be aware of the potential for Type I and Type II errors (especially Type II errors) when performing a GWAS on highly bred populations and to take appropriate countermeasures as the analysis proceeds.

### Role of *Hd1* in DTH regulation

The ETN population can be divided into early and late headings based on the *Hd1* activity. Four *Hd1* haplotypes were found in the ETN population (Figure S5A), with Hap. B is the ancestral haplotype, Hap. A, having a 36‐bp deletion in the coding region and Hap. C and D are complete loss‐of‐function haplotypes with deletions of 2 and 43 bp, respectively (Figure S5A). A comparison of the DTH of the lines carrying each of these haplotypes showed a dichotomy in Hap. A/B and Hap. C/D (Figure S14A); therefore, we treated Hap. A and Hap. B as functional haplotypes and Hap. C and Hap. D as null haplotypes. Many studies have investigated the Hap. A with a 36 bp deletion as a functional one, as well as the ancestral Hap. B (e.g., Takahashi et al., 2009; Kim et al., 2018; Wu et al., 2020). However, when comparing Hap. A and Hap. B in the ETN population, with a difference of 2.7–5.1 d depending on the year (Figure S14A). The difference was significant at the 5% level in two out of the 3 years when the difference was tested using a *t*‐test. Certain Japanese breeders have treated Hap. A as a partial loss‐of‐function type, and distinguishes it from Hap. B (Hori et al., 2023). The proportion of Hap. B decreased in the ETN population as breeding progressed and disappeared completely by Period IV (Figure S14B), suggesting that Hap. A and Hap. B was used separately for the DTH control in this panel.

### Role of *Hd16* in DTH regulation

*Hd16* encodes casein kinase I, which is a key member of the casein kinase family (Hori et al., 2016). Subsequent studies have shown that Hd16 phosphorylates and activates Ghd7 and Hd2, thereby suppressing heading in rice under certain day‐length conditions (Hori et al., 2016). Hori et al. (2016) also reported that the SNP of the “Koshihikari” *Hd16* haplotype was only observed in Japanese *japonica* cultivars and was not found in any of the wild rice, *indica* or *japonica* accessions from outside of Japan. In the present study, we re‐examined the presence of *Hd16(E)* in a larger number of lines and found that it was present in a small number of temperate *japonica* and intermediate lines from overseas (Tables S6, S7). Although it is conceivable that *Hd16(E)* found overseas could have leaked from Japan to other countries, it is more plausible that the *Hd16(E)* haplotype was originally present outside Japan, entered Japan, and subsequently spread within Japan, given that it is also found in landraces in foreign countries. Hori et al. (2016) assumed its origin to be “Moritawase,” but other landraces, such as “Kamenoo” and “Sekiyama,” also had the same haplotype (Table S6). Similar to the modern varieties carrying this haplotype, all these landraces are grown in the Tohoku region, which has historically experienced a cool climate and is often affected by cold weather damage to crops. This suggested that *Hd16(E)* has been shared and retained in some strains cultivated in this area for a long time and that this haplotype has since increased in frequency through modern breeding but has not spread to more southern or northern locations. In rice‐growing areas located above 41°N, such as northeastern China and Hokkaido, mutations in *Ghd7* and *Hd2* are important for achieving early DTH (Li et al., 2015; Fujino et al., 2019). Ghd7 and Hd2 were targets of Hd16 phosphorylation (Figure S15), and mutations in *Ghd7* and *Hd2* abolished this effect of the *Hd16* mutation. Therefore, *Hd16(E)* is probably not used in Hokkaido. However, the reason why *Hd16(E)* was not used south of 36°N could be that *Hd16(E)* has an extremely strong effect on early maturity in this area with longer summers. Many of the *Hd16(E)* lines in the ETN *Hd1* functional population also carried the late‐heading *Hd17* haplotype (Figures 4, 7), counteracting its early‐heading effect.

The present analysis also showed that *Hd16* acts as an important regulator in early (*Hd1* non‐functional) populations. This may be due to its function being (at least partially) independent of *Hd1* and its greater effect compared to that of other HD genes such as *Hd17* and *Hd18*. However, despite the potential ease of controlling DTH, *Hd16* has not yet been used for breeding outside Japan. This may be because, as mentioned above, *Hd16* is effective in the limited range of 36° to 40°N north latitude. However, as *Hd16* is an effective gene for achieving the desired DTH by breeders in this area by combining it with *Hd1*, it should be used more actively to regulate flowering time in rice outside Japan. For example, in the cultivation of temperate *japonica* varieties in northeastern China, the varieties that can be grown in each of the different temperature zones are different, and there are few varieties that can be grown across temperature zones (Heilongjiang Provincial Government, 2022). As the heading period needs to be regulated in order to straddle different temperature zones, there is great potential to apply *Hd16*‐based heading regulation to molecular breeding in northern China.

### Role of *Hd17* in DTH regulation

*Hd17* encodes a homolog of Arabidopsis ELF3 (Early Flowering 3), a transcription factor involved in the regulation of circadian rhythms; subsequent studies have shown that *Hd17*, like *Hd16*, competitively targets *Ghd7* and *Hd2* (Figure S15) (Matsubara et al., 2012). Our observations showed that there was almost no change in DTH in the active *Hd16(L)* background due to its difference, and *Hd17* can function effectively only in the case of non‐active *Hd16(E)* background (Figure 7B). Furthermore, even when we examined the interaction of *Hd16* and *Hd17* using the population where all known HD genes and polymorphisms of the peaks detected in the GWAS (Figures 2A, 3A, D) were isogenic, the effect of *Hd17* was observed only in the *Hd16(E)* background (Figure S16; Table S1). This can be interpreted as follows: Hd16 and Hd17 compete for the control of Hd2 and Ghd7 (Figure S15), the effect of Hd16 is relatively stronger than that of Hd17, and the effect of *Hd17* is greatly reduced in the functional *Hd16(L)* background, whereas the effect of *Hd17* appears in an inactive *Hd16(E)* background. Indeed, in the transition of haplotype frequencies of *Hd16* and *Hd17* during the breeding process of the *Hd1* functional population, the late (active) to early (inactive) transition of *Hd16* occurred from Periods I to II, but not from *Hd17*, whereas after the inactive *Hd16(E)* lines became dominant, *Hd17(L)* selection occurred from Periods II to III (Figure 4). This is in good agreement with the observation that only the *Hd16(E)* background could distinguish the *Hd17* haplotype from DTH.

### Role of *Hd18* in DTH regulation

In the early‐heading population, most of which had non‐functional *Hd1* haplotypes, *Hd16* and *Hd18* controlled DTH between 60 and 70 d, and this range was much smaller than that in the late‐heading population (Figure S13). We examined the interaction between the effects of *Hd16* and *Hd18* and found that the functional *Hd16 (L)* lines (red dots in Figure 7D–F) tended to show a greater effect of *Hd18* than the non‐functional *Hd16(E)* backgrounds (blue dots). In a study using the near isogenic lines (NILs) with the Tohoku206 background, the effect of each combination of the *Hd1, Hd16*, and *Hd18* haplotypes on DTH was reported (Ishimori et al., 2020; summarized in Figure S17A). Using these NILs, we investigated whether the effect of *Hd18* on DTH differed in the *Hd16(E)* and *Hd16(L)* backgrounds and observed that the effect of *Hd18* was stronger in the *Hd16(L)* than *Hd16(E)* background (Figure S17B, C). Hd16 inhibited Ehd1 function via Ghd7, whereas *Hd18* positively regulated *Ehd1* (Figure S15). In the case of functional *Hd16(L)*, Ehd1 activity was repressed by *Ghd7*, whereas in non‐functional *Hd16(E)*, *Ehd1* function was expected to be in a liberated state. Assuming that Hd18 releases Ehd1 activity in the repressed state, it is reasonable that its effect on Ehd1 is greater in the repressed state than in the released state. However, in contrast to the late‐heading population, there was no evidence of selection for specific DTH during the breeding process (Figure S13D, E). The early‐heading varieties in Fukui were inferior to the late‐heading varieties in terms of protein content (Figure 5B). However, from an industrial perspective, early‐heading varieties occupy an important position in rice production in Fukui Prefecture, as mentioned above. Under these circumstances, early‐heading varieties are required to carefully control DTH. In this context, the use of *Hd16* and *Hd18* succeeded in ensuring DTH variation between 60 and 70 d, as the use of non‐functional *Hd1* narrowed the range of DTH to achieve early heading.

## MATERIALS AND METHODS

### Plant materials

Of the 275 Etsunan lines from ETN001 to ETN305 bred at the FAES from 1949 to 2019, 252 lines were tested, excluding glutinous rice, rice with low and high amylose content, aromatic rice, rice suitable for sake brewing, and powdery rice. Seeds were from the breeder's seeds frozen and stored at FAES; ETN019, ETN084, and ETN089 seeds were obtained from NARO Genebank (https://www.gene.affrc.go.jp/index_en.php). Each line was classified as early (including very early and early late), medium, or late heading according to the breeding reports of new paddy rice lines (Fukui Agricultural Experiment Station, 1950–2020) prepared at each breeding time.

Twenty seedlings of each line were transplanted into the paddy field at FAES from May 17 to 21 in 2019–2021 and cultivated with 6.6 kg nitrogen/10a of basal fertilizer. The day length, average temperature, and sunshine duration during the paddy rice‐growing season in Fukui Prefecture are shown in Figure S18. The day on which more than half of the individuals headed was defined as the heading date, and the period from the transplantation date to the heading date was defined as the DTH. Ten mature individuals were harvested and examined for culm length and panicle weight. After drying and hulling, the protein content in brown rice was measured using a rice analyzer TM‐3500 (Shizuoka Seiki Co. Ltd., Shizuoka, Japan), and the amylose content in polished rice was measured using an Auto Analyzer III (BL TEC K. K., Osaka, Japan).

### Four periods in rice breeding in Fukui

The history of rice breeding at the FAES can be divided into four periods according to the main breeding objectives and selection methods, as shown in Table 1.

Period I (1947–1969): High yield and disease resistance were the main breeding goals, because food production increase was the goal for all of Japan in the post‐World War II period. It includes 93 ETN lines from ETN001 to ETN099.

Period II (1970–1992): High eating quality was added to the main breeding objective, and selection based on sensory tests for eating quality began. Reconstruction from WWII progressed in addition to abundant harvests in the late 1960s, leading to excess rice inventory, which brought the rice distribution system not subject to government price control since 1969, and the need for rice changed drastically from quantity to quality. Improvements in the yield and disease resistance have continued. It includes 55 ETN lines, from ETN100 to ETN157.

Period III (1993–2005): High eating quality was the main breeding objective; however, selection based on the amylose content in the endosperm was added. Research on rice‐eating quality has progressed, and it is clear that a low amylose content in the endosperm is important for high eating quality (Okuno et al., 1983; Inatsu, 1988; Yano et al., 1988). Selection using sensory tests for eating quality, improvements in yield, and disease resistance were continued. It includes 54 ETN lines, from ETN158 to ETN210.

Period IV (2006–2019): From around 2000, high temperatures during the ripening period reduced the rice quality in Fukui Prefecture (Kobayashi, 2012). Tolerance to heat‐induced quality decline during ripening was added to the main breeding objective, and selection by apparent grain quality ripening in a high‐temperature greenhouse was added. Selection was made using sensory tests for eating quality, and improvements in yield, and disease resistance were continued. It includes 50 ETN lines, from ETN211 to ETN305.

### Genotyping

DNA preparation and genotyping were performed as previously described (Suganami et al., 2024). DNA for genotyping was isolated from the leaves using a DNeasy Plant Mini Kit (#69104; Qiagen, Hilden, Germany) and fragmented to approximately 500 bp using Covaris S2 (Covaris, Brighton, UK). NEBNext DNA Library Prep Reagent Set (New England Biolabs, Ipswich, MA, USA, #E6000) was used to construct a DNA library. Paired‐end sequencing was performed using an Illumina HiSeq system (Illumina Co. Ltd., San Diego, CA, USA) with a read length of 100–150 bp. All reads were mapped against Os‐NipponbareReference‐IRGSP‐1.0 pseudomolecules (all.con ver.7, downloaded from http://rice.plantbiology.msu.edu/pub/data/Eukaryotic_Projects/o_sativa/annotation_dbs/pseudomolecules/version_7.0/all.dir/), and fastq files were converted into samfiles using the bwa‐mem command of BWA software ver0.7.18. Commands samtools‐view, samtools‐sort, and samtools‐index of Samtools software ver1.6 were used to successively generate, sort, and index the BAM files. The variants for each accession were called using the GATK HaplotypeCaller (release 4.0.4.0) with the “.g.vcf” extension. The GATK Genomics DB Import and Genotype GVCFs were used for joint genotyping to produce a single variant call format (VCF) file from per‐sample GVCF files.

SnpEff software version 4.3T (Cingolani et al., 2012) was used to predict the effects of genomic variants on gene function. General feature format version 3 (gff3) from the Rice Genome Annotation Project (Ouyang et al., 2007; http://rice.plantbiology.msu.edu/) was used to provide information on gene position and coding sequences. For GWAS analysis, SNPs with minor allele frequencies (<5%) and missing rate (≥10%) were filtered out.

### Population genetic analyses and GWAS

The population structure was estimated using PCA performed by using the R package “SNPRelate” version 4.2 (Zheng et al., 2012). We used a linear mixed model (LMM) for the GWAS. Genome‐wide association studies were performed using the R packages “rrBULP” version 4.3, according to Yano et al. (2016). Genome‐wide thresholds were set to the significance threshold (*P* = 5.0 × 10^−8^; red) and suggestive threshold (*P* = 1.0 × 10^−5^; blue), which is the default of rrBLUP. Fixed effects such as principal components were not included in the calculation. We performed a GWAS using the polymorphism with the highest signal in the targeted peak region as a covariate to determine whether the peak had disappeared (Suganami et al., 2023). The disappearance of a peak indicates that its explanatory potential was removed by the fixation effect (Segura et al., 2012), and the vanished peak was judged to be in linkage disequilibrium with the polymorphism used as a covariate. Nei's genetic distance was calculated for Periods I and IV varieties using an in‐house script (Yano et al., 2019), and the thresholds were set at the top 5% and 10% of the genetic distance for each chromosome.

### Correlation and partial correlation analysis

A correlation analysis between genotypes and DTH was conducted to determine the effect of haplotypes of HD genes on DTH in the ETN lines. Partial correlation analysis was also conducted to distinguish true correlations from false correlations caused by the effects of other correlated genes. Among the 22 known HD genes (Wei et al., 2021; Table S2), polymorphisms were found in ten genes and six genes (*Hd1*, *Hd2*, *Hd6*, *Hd16*, *Hd17*, and *Hd18*) were present at frequencies of 5% or higher. The haplotypes of six genes were coded 0 for lower activity and 1 for higher activity. The associations between the six genotypes and DTH were examined using point‐biserial correlation coefficients and partial correlation analyses. The phi coefficient, a measure of the association between two binary variables, was used to determine the correlation between the HD genes. As these coefficients are the same as Pearson's product‐rate correlation coefficient when one or both variable pairs are binary variables, it is convenient to use them in a partial correlation analysis.

## CONFLICTS OF INTEREST

The authors declare no conflict of interest.

## AUTHOR CONTRIBUTIONS

A.K. and M.M. conceived the research plan. A.K., S.W., Y.M., G.C., F.N., and N.S. managed the cultivation and evaluated traits of ETN lines. H.Y. and K.M. performed the genotyping. M.S. performed the GWAS and population genetics analyses. Y.M. performed correlation and partial correlation analyses. A.K., M.S., H.Y., and M.M. wrote and edited the manuscript. All authors have approved the submitted version of the manuscript.

## Supporting information

Additional Supporting Information may be found online in the supporting information tab for this article: http://onlinelibrary.wiley.com/doi/10.1111/jipb.13785/suppinfo

**Figure S1.** Genetic population structure of ETN lines
**Figure S2.** Correlation between PC scores and DTH
**Figure S3.** GWAS for DTH in 252 overall ETN population in 2019 **(A**–**D)** and 2021 **(E**–**H)**
**Figure S4.** Quantile–quantile plots of GWAS of DTH of ETN lines
**Figure S5.** Exon‐intron structure of *Hd1* **(A)**, *Hd16* **(B)**, *Hd6* **(C)**, *Hd18* **(D)**, *Hd2* **(E)** and *Hd17* **(F)** with DNA polymorphisms reported to disrupt gene function
**Figure S6.** Local Manhattan plots of GWAS for DTH in 252 overall ETN population in 2020
**Figure S7.** GWAS for DTH in 114 early‐heading and 138 late‐heading lines in 2019 and 2021
**Figure S8.** Haplotype frequency of *Hd1* in 114 early‐heading **(A)** or 138 late‐heading ETN lines **(B)**
**Figure S9.** Local Manhattan plot of GWAS for DTH in 138 late‐heading lines surrounding Peak 7 (7–10 Mb on Chr. 6)
**Figure S10.** GWAS for DTH in *Hd1* functional and non‐functional groups
**Figure S11.** Nei's genetic distance between the breeding period I and IV in *Hd1* functional population
**Figure S12.** Variation in DTH in *Hd1* functional population by combinations of *Hd16* and *Hd17* haplotypes
**Figure S13.** Variation in DTH of ETN lines with non‐functional *Hd1* (Hap C and Hap D) by genotypic combinations of two flowering‐related genes, *Hd16* and *Hd18*
**Figure S14.** Characteristic of *Hd1* haplotype
**Figure S15.** Gene Networks explaining *Hd16, Hd17*, and *Hd18* interactions
**Figure S16.** Gene‐by‐gene (GxG) interaction of *Hd16* and *Hd17* in the population with same genetic background
**Figure S17.** Gene‐by‐gene (GxG) interaction of *Hd16* and *Hd18* using NIL lines of Tohoku 206 and Koshihikari reported in Ishimori et al. (2020)
**Figure S18.** Environmental condition in Fukui

**Table S1.** Genomic and phenotypic data of Etsunan lines
**Table S2.** DNA polymorphisms of the 22 HD genes
**Table S3.** Matrix of the phi coefficients between six heading genes in the overall ETN population
**Table S4.** Matrix of the phi coefficients between five heading genes in the Hd1 non‐functional population
**Table S5.** Matrix of the phi coefficients between five heading genes in the Hd1 functional population
**Table S6.** List of Japanese landraces and foreign strains with Hd16(E)
**Table S7.** Allele frequencies in Hd16 in rice variation map v2.0
