# Supplementary material for: How have breeders adapted rice flowering to the growing region?
Source: J Integr Plant Biol. 2024 Oct 25;66(12):2736–53. doi: 10.1111/jipb.13785 (PMC11622534; doi:10.1111/jipb.13785)
Supplement: Supplementary file 1 — Figure S1. Genetic population structure of ETN lines Figure S2. Correlation between PC scores and DTH Figure S3. GWAS for DTH in 252 overall ETN population in 2019 (A–D) and 2021 (E–H) Figure S4. Quantile–quantile plots of GWAS of DTH of ETN lines Figure S5. Exon‐intron structure of Hd1 (A), Hd16 (B), Hd6 (C), Hd18 (D), Hd2 (E) and Hd17 (F) with DNA polymorphisms reported to disrupt gene function Figure S6. Local Manhattan plots of GWAS for DTH in 252 overall ETN population in 2020 Figure S7. GWAS for DTH in 114 early‐heading and 138 late‐heading lines in 2019 and 2021 Figure S8. Haplotype frequency of Hd1 in 114 early‐heading (A) or 138 late‐heading ETN lines (B) Figure S9. Local Manhattan plot of GWAS for DTH in 138 late‐heading lines surrounding Peak 7 (7–10 Mb on Chr. 6) Figure S10. GWAS for DTH in Hd1 functional and non‐functional groups Figure S11. Nei's genetic distance between the breeding period I and IV in Hd1 functional population Figure S12. Variation in DTH in Hd1 functional population by combinations of Hd16 and Hd17 haplotypes Figure S13. Variation in DTH of ETN lines with non‐functional Hd1 (Hap C and Hap D) by genotypic combinations of two flowering‐related genes, Hd16 and Hd18 Figure S14. Characteristic of Hd1 haplotype Figure S15. Gene Networks explaining Hd16, Hd17, and Hd18 interactions Figure S16. Gene‐by‐gene (GxG) interaction of Hd16 and Hd17 in the population with same genetic background Figure S17. Gene‐by‐gene (GxG) interaction of Hd16 and Hd18 using NIL lines of Tohoku 206 and Koshihikari reported in Ishimori et al. (2020) Figure S18. Environmental condition in Fukui [file JIPB-66-2736-s002.pdf]

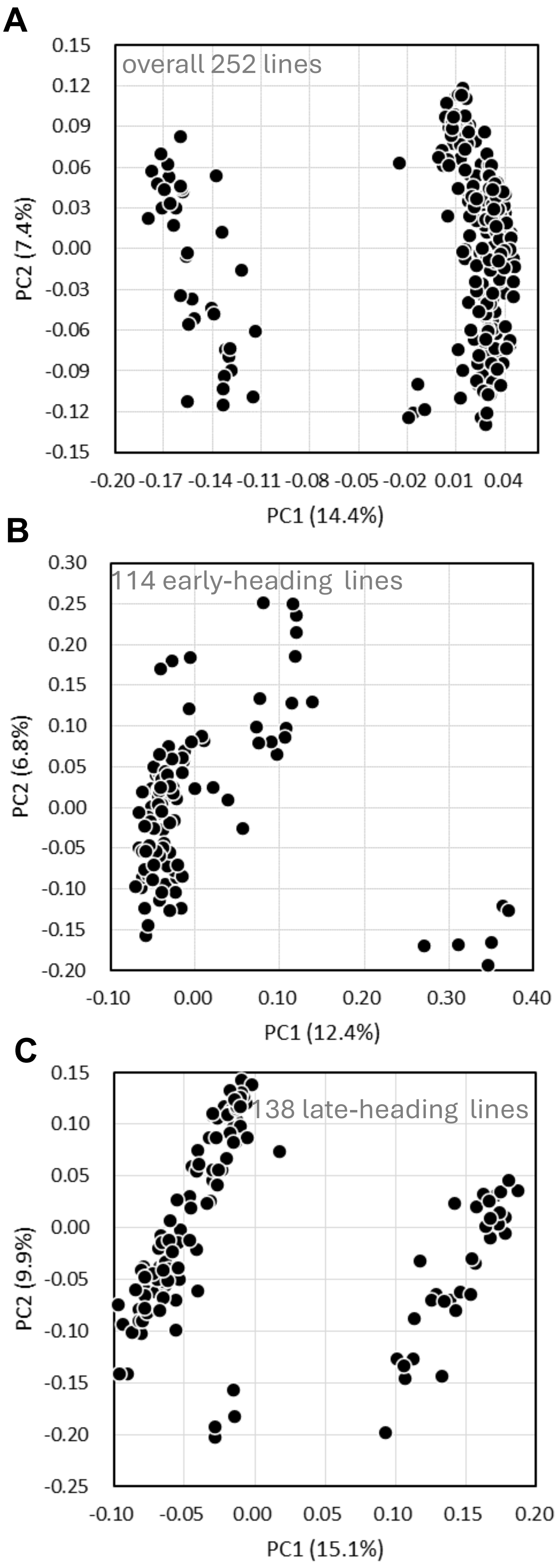

Figure S1. Genetic population structure of ETN lines.
Principal component analyses for (A): overall 252 lines, (B): 114 early-heading lines, and (C): 138 late-heading lines. PC1 and PC2 indicate the score of principal components 1 and 2, respectively. Values in parentheses indicate percentage of variance in the data explained by each principal component.

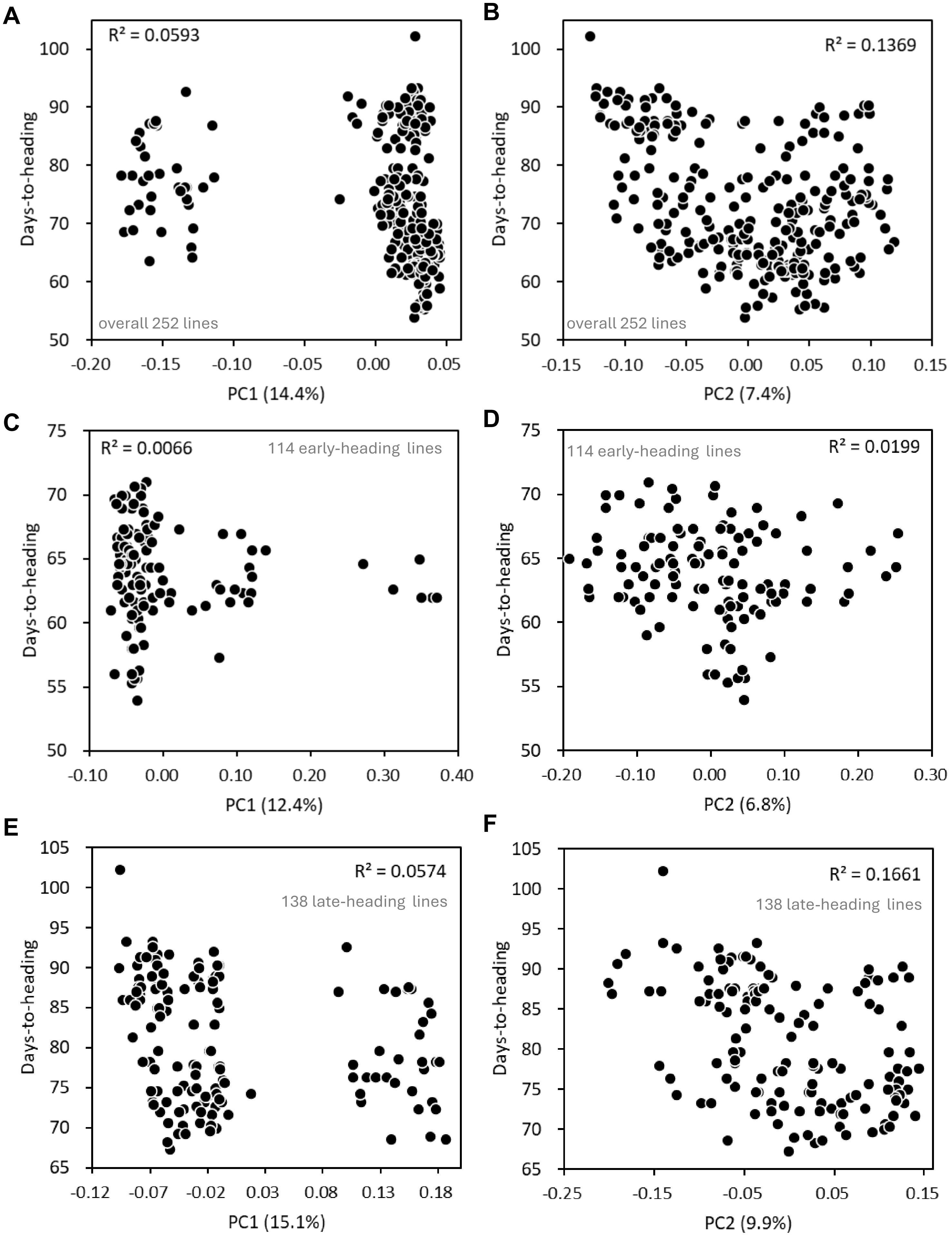

Figure S2. Correlation between PC scores and DTH.
Correlation between DTH and PC1 (A, C, E) and PC2 (B, D, F) in overall 252 lines (A, B), 114 early-heading lines (C, D), 138 late-heading lines (E, F).

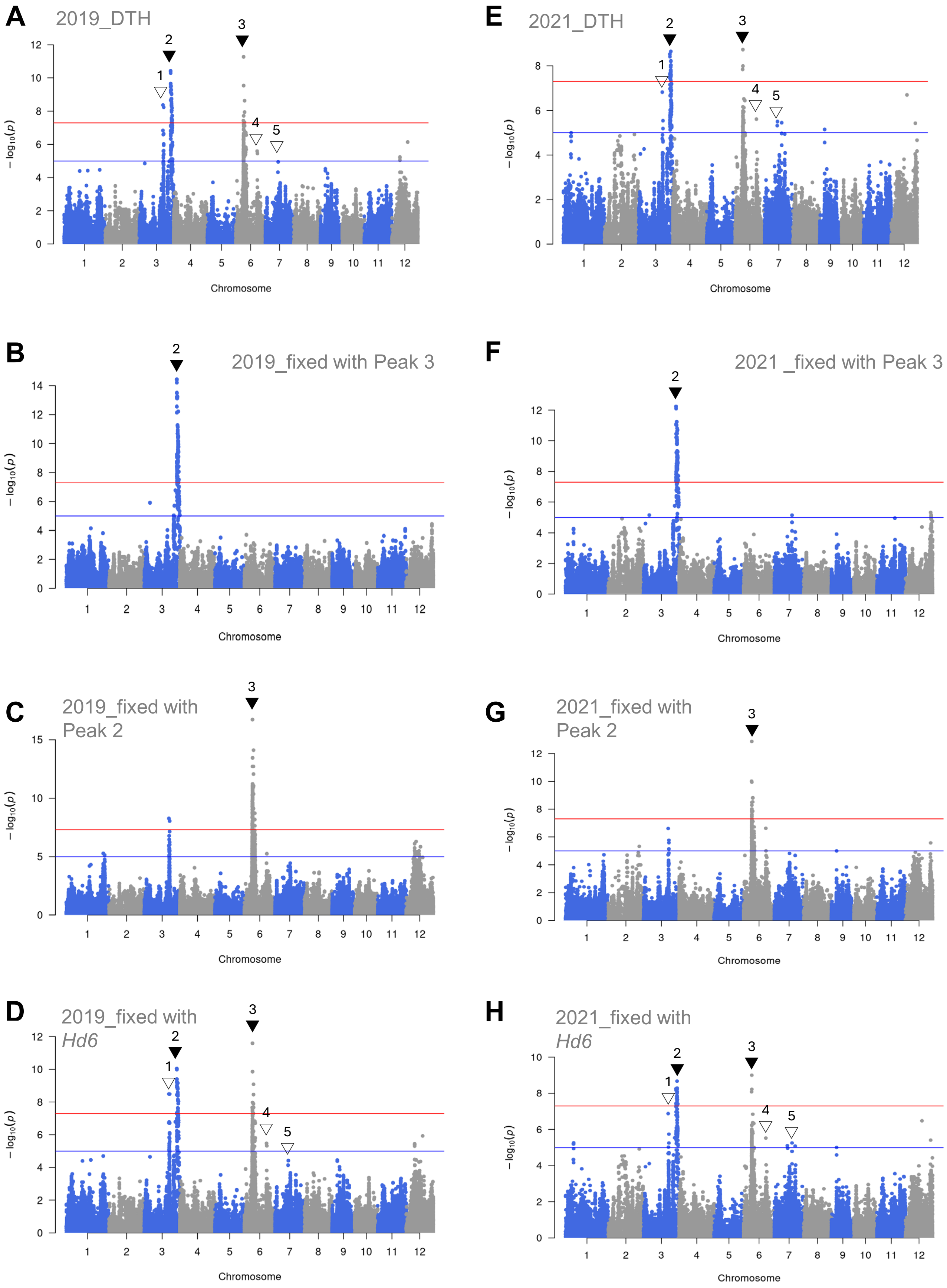

Figure S3. GWAS for DTH in 252 overall ETN population in 2019 (A-D) and 2021 (E-H).
(A, E) Manhattan plot of GWAS for DTH in 2019 (A) and 2021 (E). (B-D, F-H) Manhattan plot of GWAS using the polymorphism with the highest $-\log_{10}(P)$ within Peak 3 (B, F) and Peak 2 (C, G), and using the causative polymorphism of *Hd6* (D, H). Genome-wide thresholds were set to the significance threshold ($P = 5.0 \times 10^{-8}$; red) and suggestive threshold ($P = 1.0 \times 10^{-5}$; blue). Arrowheads indicate the peaks that are above threshold for all three years 2019, 2020, and 2021. Black arrowheads indicate the peaks corresponding to known HD genes and white arrowheads indicate the peaks considered to be false positive.

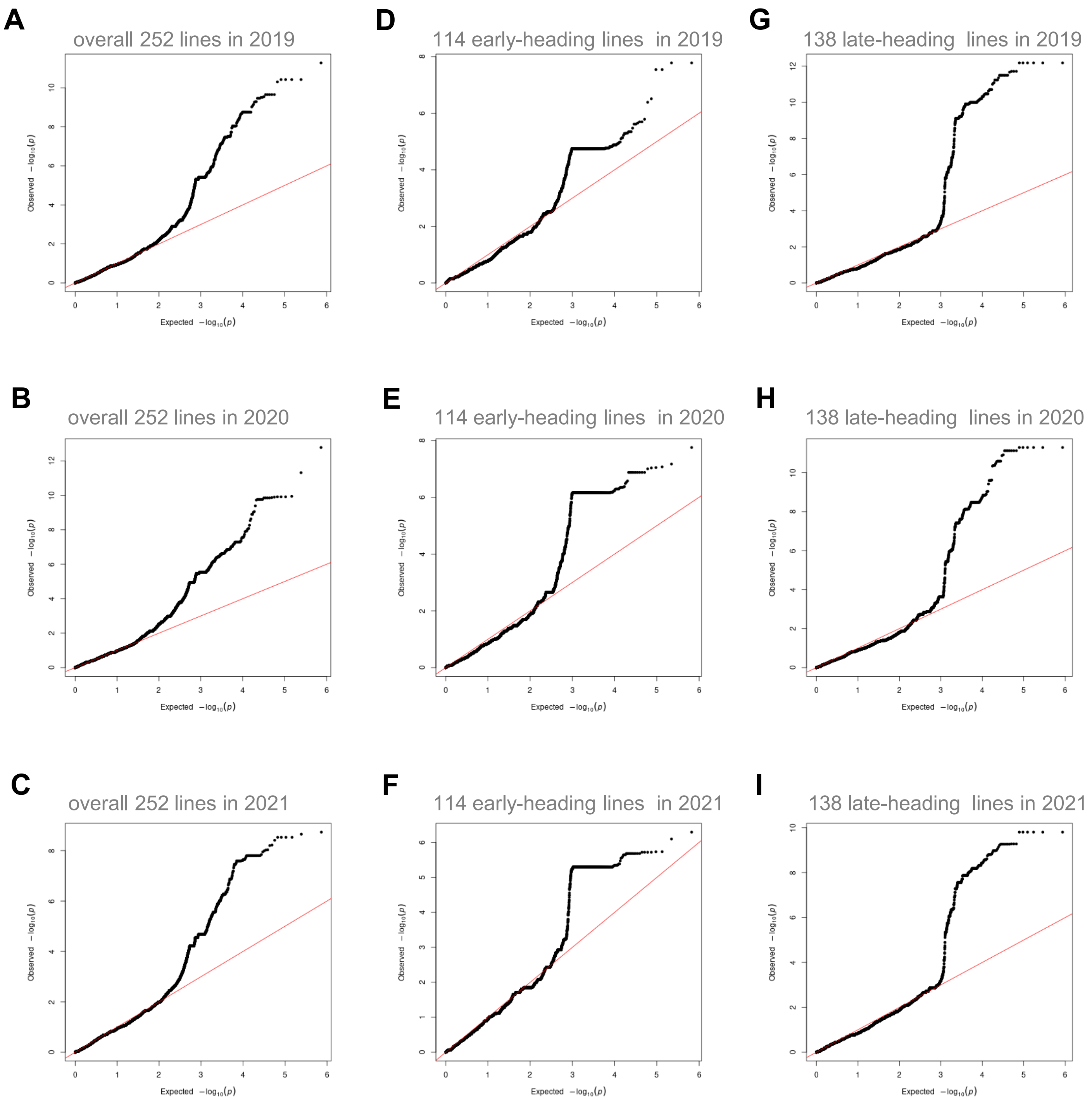

Figure S4. Quantile-quantile plots of GWAS of DTH of ETN lines.
The red line represents an ideal case where theoretical test statistic quantiles match the simulated test statistic quantiles.

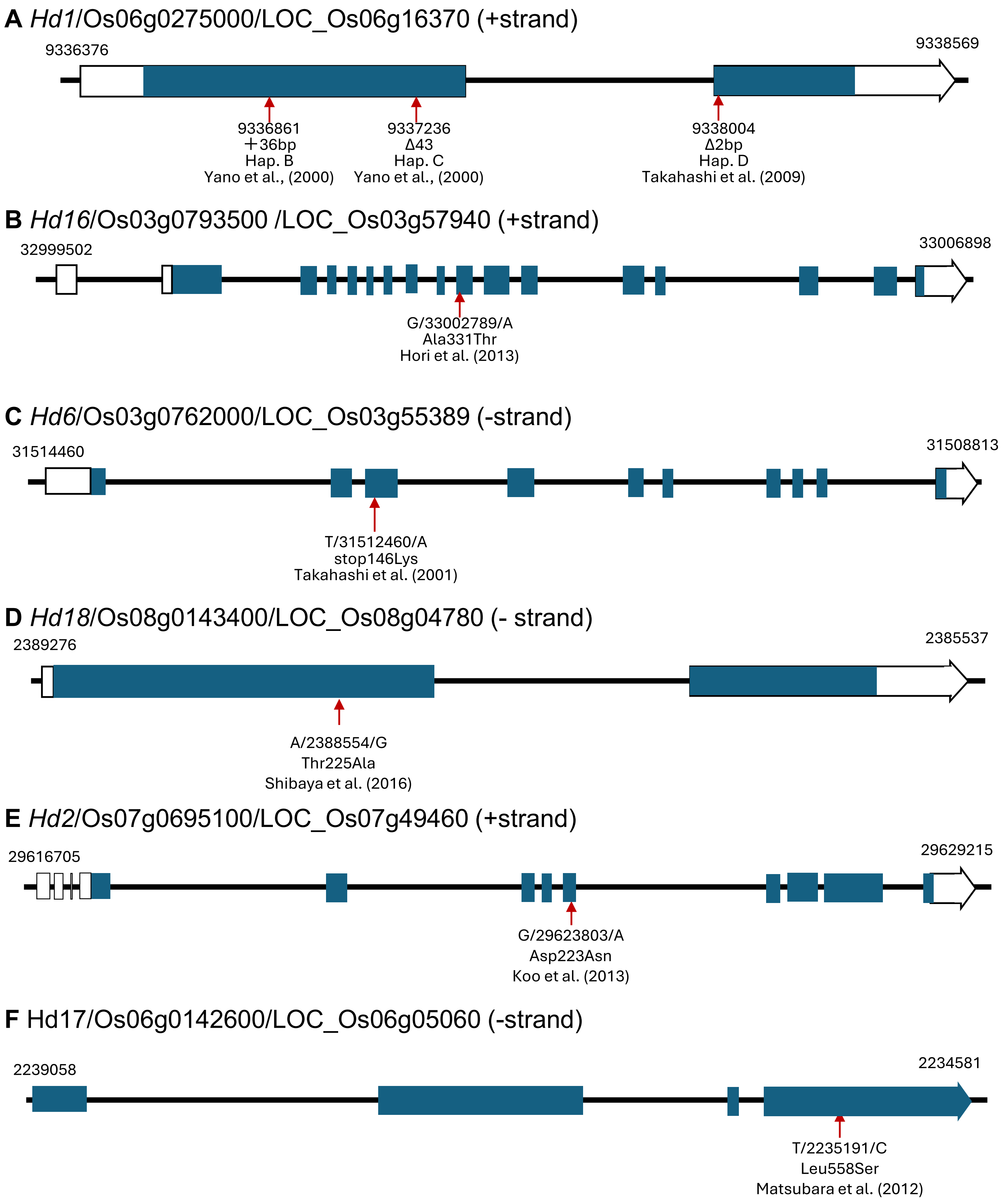

Figure S5. Exon-intron structure of *Hd1* (A), *Hd16* (B), *Hd6* (C), *Hd18* (D), *Hd2* (E) and *Hd17* (F) with DNA polymorphisms reported to disrupt gene function.

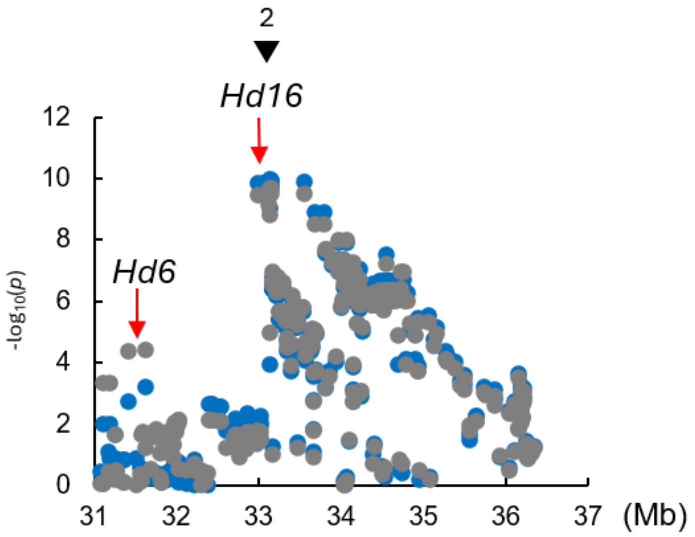

Figure S6. Local Manhattan plots of GWAS for DTH in 252 overall ETN population in 2020.

Local Manhattan plot of GWAS surrounding the Peak 2 (31-37 Mb on Chr. 3). The red arrow indicate the position of *Hd6* and *Hd16*. Plots shows the results of GWAS performed without (blue) or with (gray) the causative polymorphism of *Hd6* .

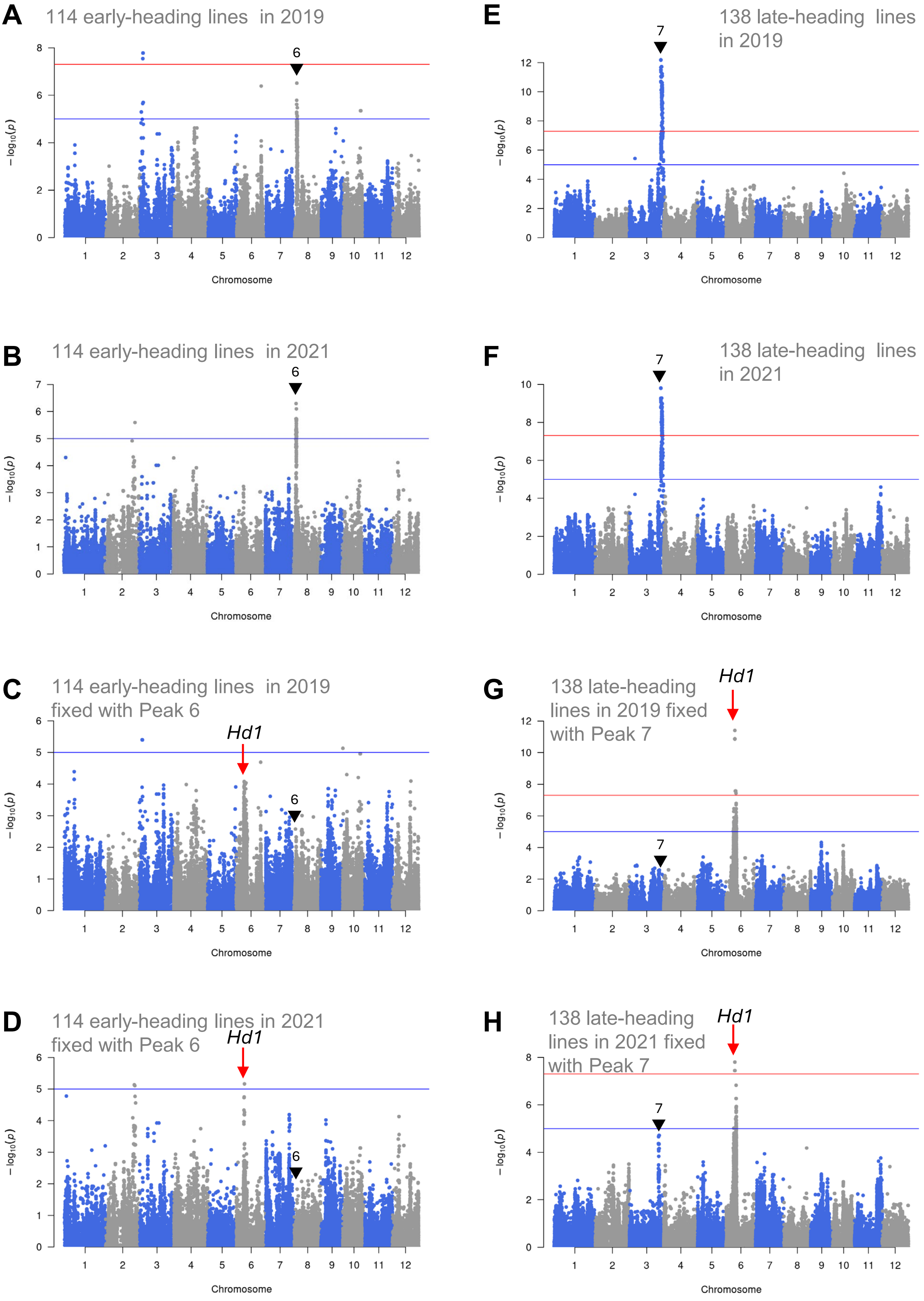

Figure S7. GWAS for DTH in 114 early-heading and 138 late-heading lines in 2019 and 2021. (A, B, E, F) Manhattan plot of GWAS for DTH in 114 early-heading (A, B) and 138 late-heading lines (E, F) in 2019 (A, E) and 2021 (B, F). (C, D, G, H) Manhattan plot of GWAS using the polymorphism with the highest $-\log_{10}(P)$ within Peak 6 (C, D) and Peak 7 (G, H). Genome-wide thresholds were set to the significance threshold (P = 5.0 x 10^{-8}; red) and suggestive threshold (P = 1.0 x 10^{-5}; blue). Black arrowheads indicate the peaks that are above threshold for all three years 2019, 2020, and 2021. The red arrow indicate the position of *Hd1* genes.

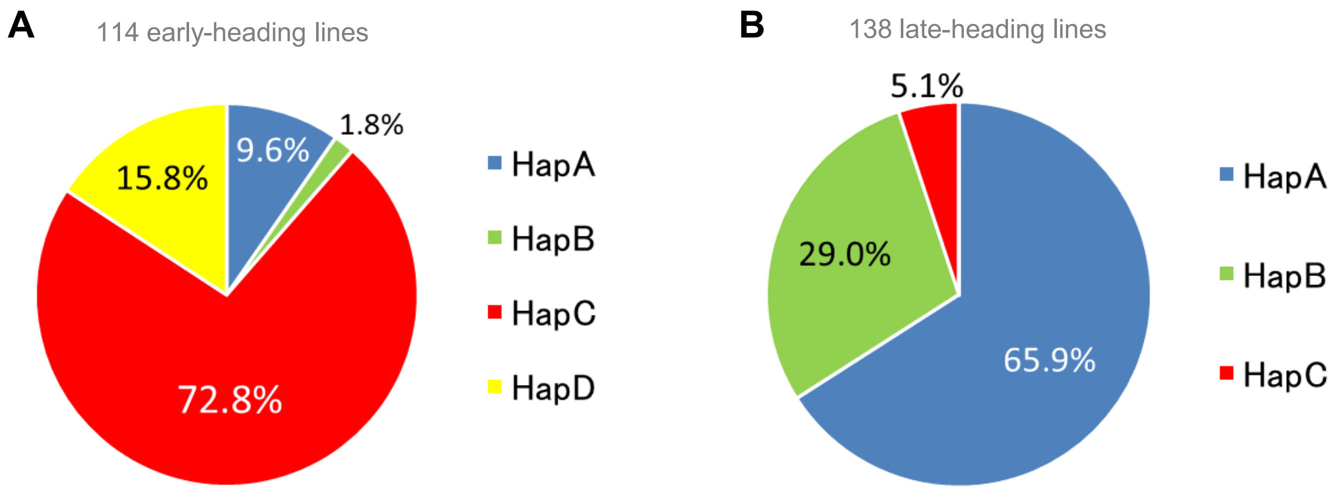

Figure S8. Haplotype frequency of *Hd1* in 114 early-heading (A) or 138 late-heading ETN lines (B).

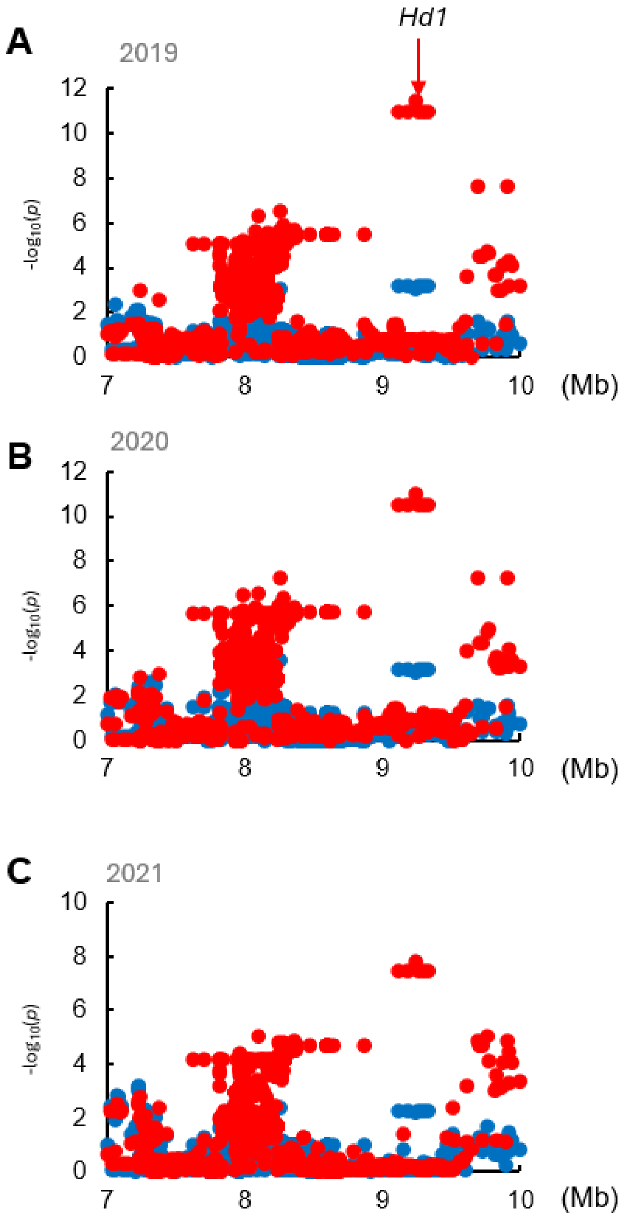

Figure S9. Local Manhattan plot of GWAS for DTH in 138 late-heading lines surrounding Peak 7 (7-10 Mb on chr. 6). The red arrow indicate the position of *Hd1*. Plots shows the results of GWAS performed without (blue; A-C) or with (red) the polymorphism with the highest -$\log_{10}(P)$ within the peak region.

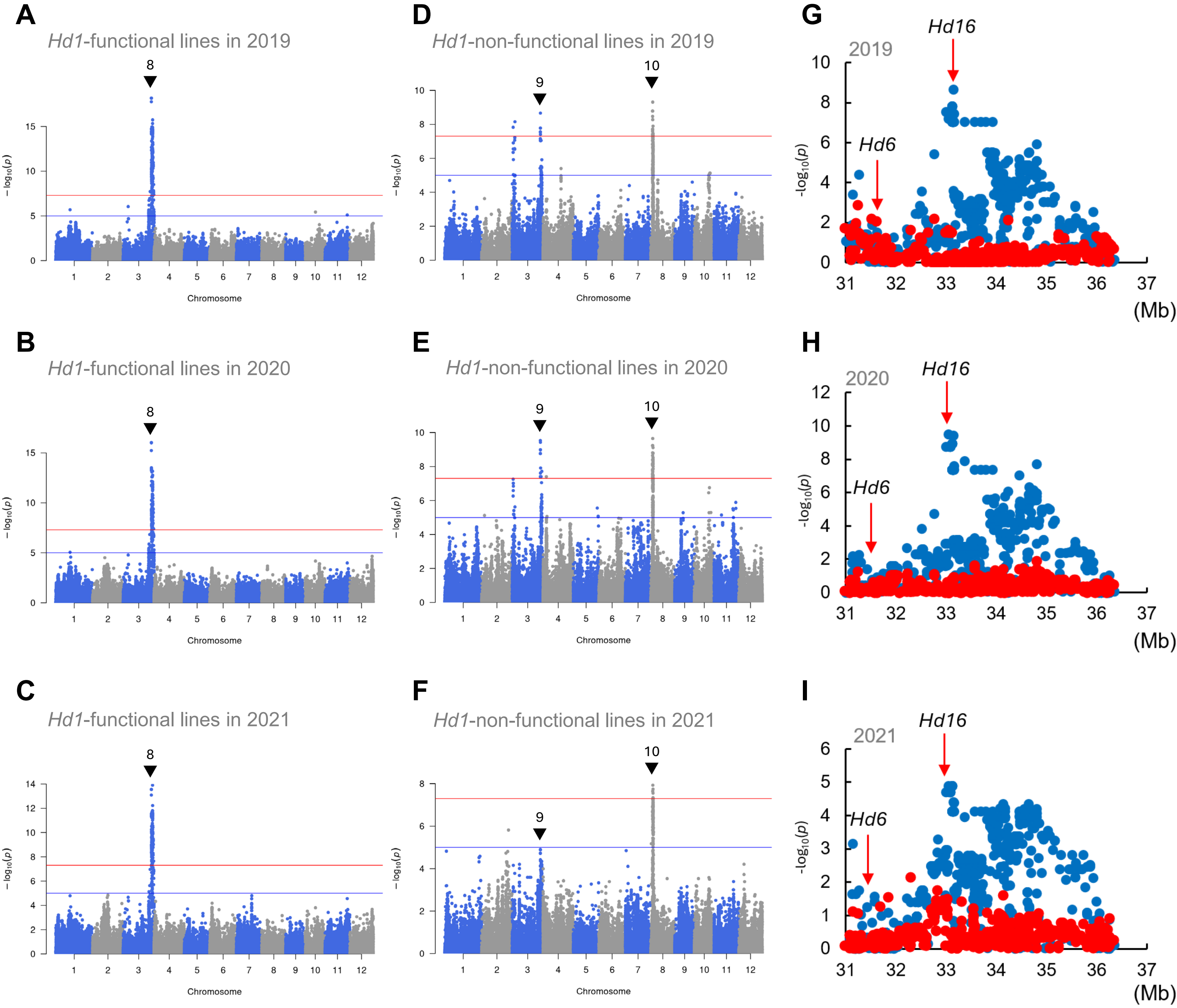

Figure S10. GWAS for DTH in *Hd1* functional and non-functional groups.
(A-F) Manhattan plot of GWAS for DTH in 144 *Hd1*-functional (A-C) and 108 *Hd1*-non-functional lines (D-F) in 2019 (A, D), 2020 (B, E) and 2021 (C, F). Genome-wide thresholds were set to the significance threshold (P = 5.0 x 10^{-8}; red) and suggestive threshold (P = 1.0 x 10^{-5}; blue). Black arrowheads indicate the peaks that corresponds to *Hd16* and *Hd18*. (G-F) Local Manhattan plot surrounding Peak 9 (7-10 Mb on Chr. 6). The red arrow indicate the position of *Hd6* and *Hd16*. Plots shows the results of GWAS performed without (blue) or with (red) the polymorphism with the highest $-\log_{10}(P)$ within the peak region.

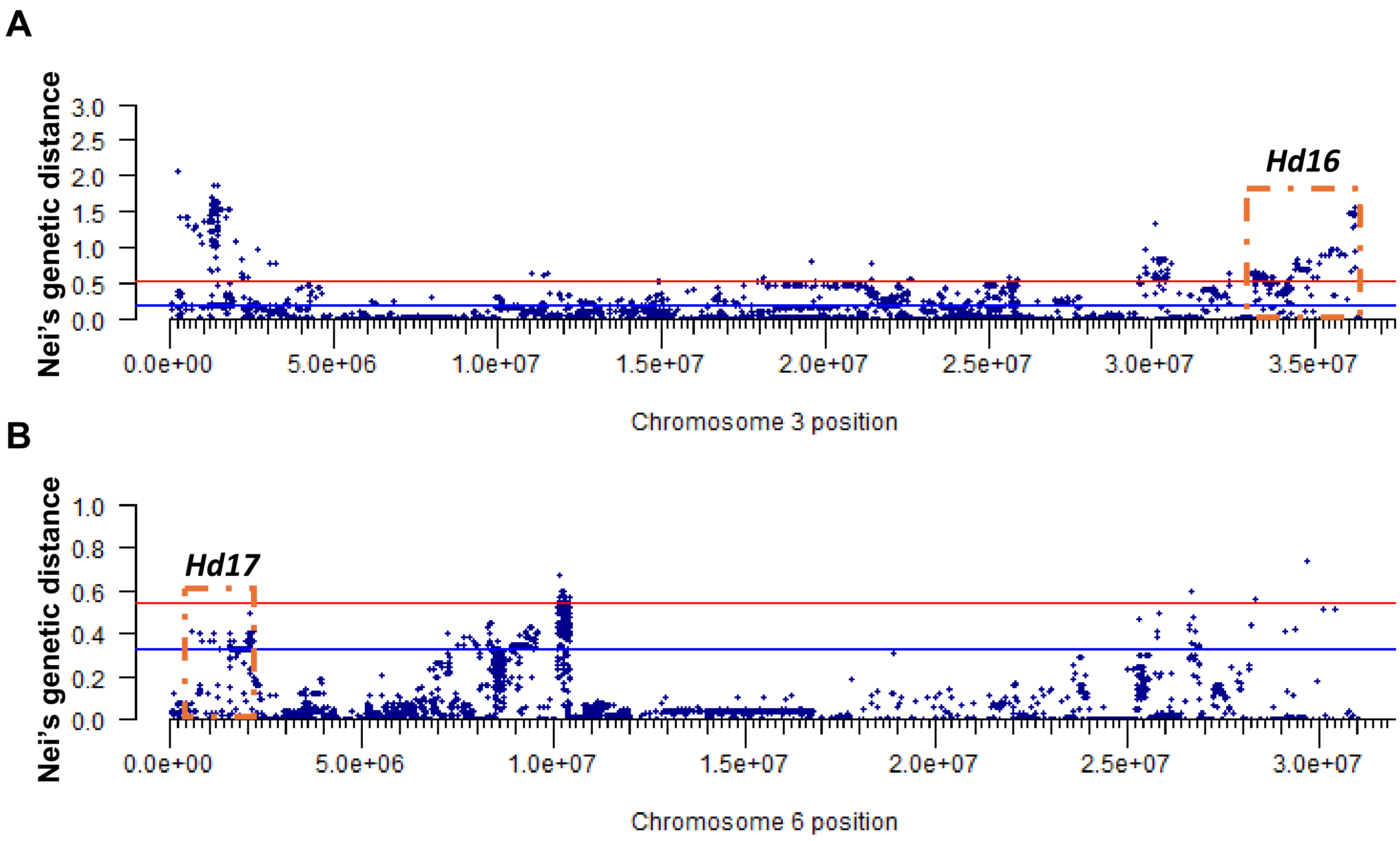

Figure S11. Nei's genetic distance between the breeding period I and IV in *Hd1* functional population.

(A, B) Semi local plot of Nei's genetic distance on Chr. 3 (A) and Chr. 6 (B). Thresholds were set 5% (red) and 10% (blue) for each chromosome. The orange dashed squares indicate peak regions including *Hd16* and *Hd17*.

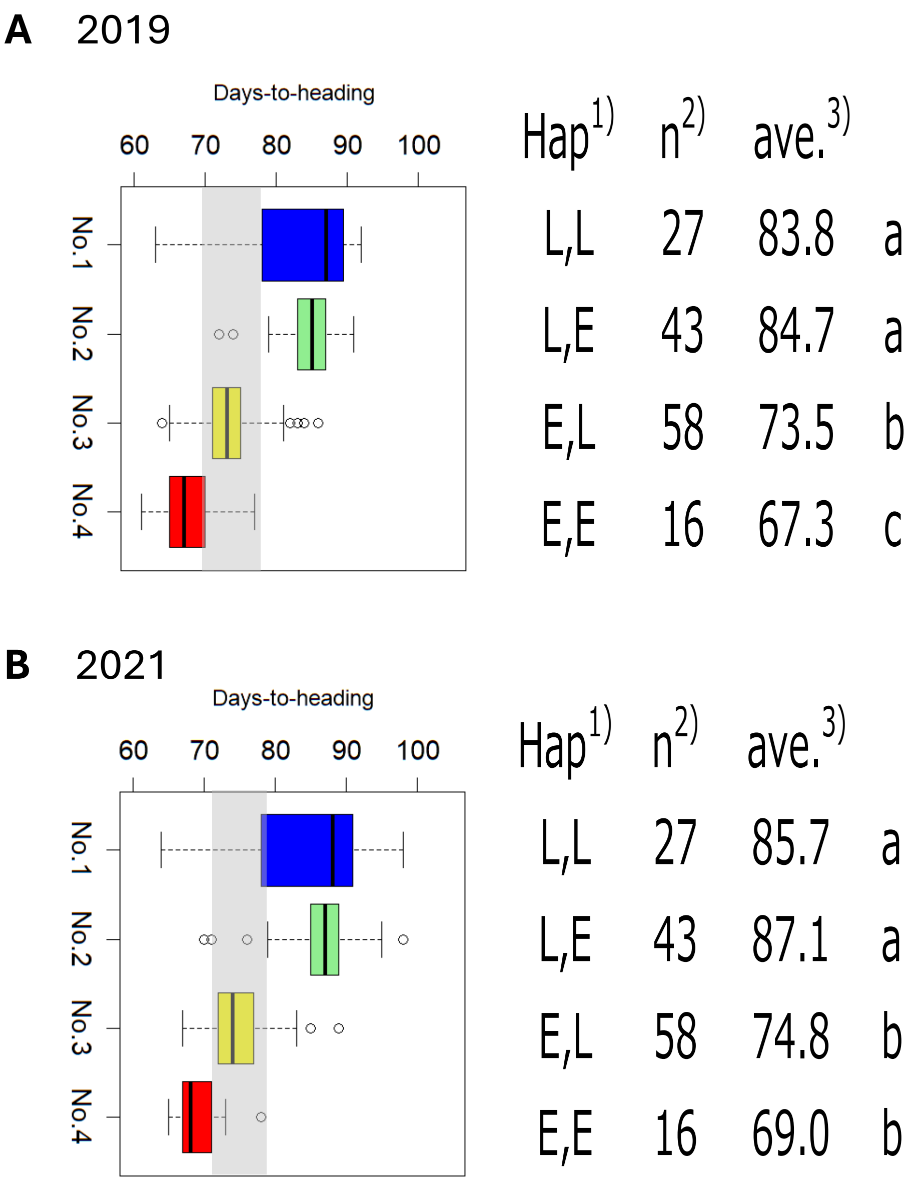

Figure S12. Variation in DTH in *Hd1* functional population by combinations of *Hd16* and *Hd17* haplotypes.

(A, B) Boxplot of DTH for each group classified by haplotype combination of heading in 2019 (A) and 2021 (B). 1) Haplotype of *Hd16*, *Hd17*, L: Later heading haplotype, E: Earlier heading haplotype. 2) Number of lines. 3) Means denoted by a different letter indicate significant differences at p-value <0.05 by Tukey-Kramer multiple comparison. The gray area of days-to-heading shows the average ±SD of DTH in 2019 (A) and 2021 (B) induced by the haplotype No.3.

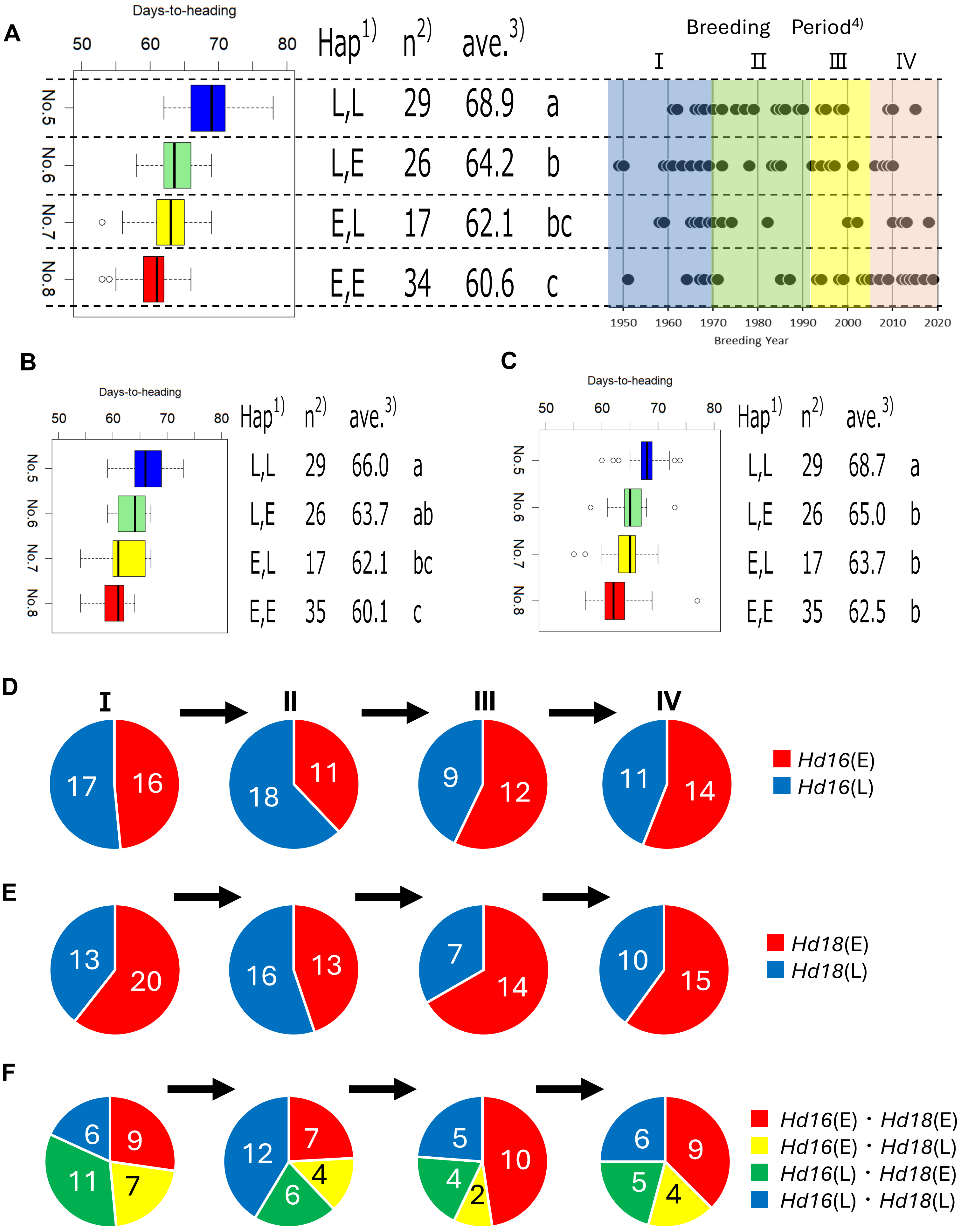

A
Days-to-heading
50 60 70 80
Hap1) n2) ave.3)
No.5 L,L 29 68.9 a
No.6 L,E 26 64.2 b
No.7 E,L 17 62.1 bc
No.8 E,E 34 60.6 c
Breeding Period4)
I II III IV
1950 1960 1970 1980 1990 2000 2010 2020
Breeding Year
B
Days-to-heading
50 60 70 80
Hap1) n2) ave.3)
No.5 L,L 29 66.0 a
No.6 L,E 26 63.7 ab
No.7 E,L 17 62.1 bc
No.8 E,E 35 60.1 c
C
Days-to-heading
50 60 70 80
Hap1) n2) ave.3)
No.5 L,L 29 68.7 a
No.6 L,E 26 65.0 b
No.7 E,L 17 63.7 b
No.8 E,E 35 62.5 b
D
I II III IV
17 16
18 11
9 12
11 14
Hd16(E)
Hd16(L)
E
13 20
16 13
7 14
10 15
Hd18(E)
Hd18(L)
F
6 9 11 7
12 7 6 4
5 10 4 2
6 9 5 4
Hd16(E)・Hd18(E)
Hd16(E)・Hd18(L)
Hd16(L)・Hd18(E)
Hd16(L)・Hd18(L)

Figure S13. Variation in DTH of ETN lines with non-functional *Hd1* (Hap C and Hap D) by genotypic combinations of two flowering-related genes, *Hd16* and *Hd18*.

(A) Boxplot of DTH in 2020 for each group classified by haplotype combination of flowering-related genes, and the breeding year of the ETN lines belonging to each group. 1) Haplotype of *Hd16* and *Hd18*, L: Later heading allele, E: Earlier heading allele. 2) Number of lines. 3) Means denoted by a different letter indicate significant differences at 5% level by Tukey-Kramer multiple comparison. 4) See Table 1. (B) and (C) Boxplot of DTH in 2019 and 2021, respectively. (D-F) Haplotype ratio of *Hd16* (D) and *Hd18* (E), and ratio of '*Hd16*・*Hd18*' combination (F) during breeding process, Period I to IV (see Table 1). "E" means earlier heading haplotype, "L" means later heading haplotype. Number inside the circle represents number of lines. There was no significant change at 5% level in a Fisher's exact test that allows to reject the hypothesis that the ratio of haplotypes or polymorphisms in one period was maintained in the next period.

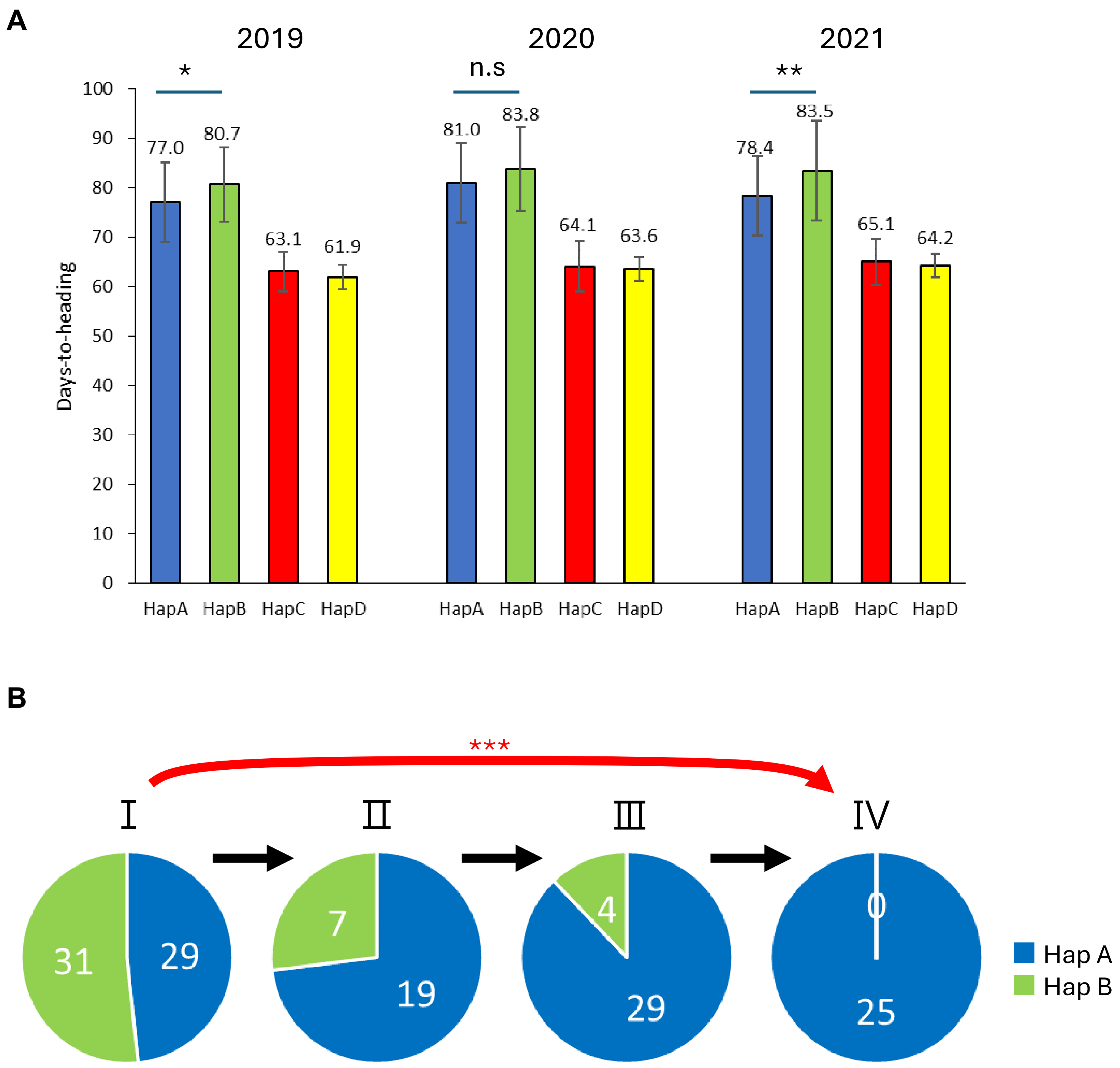

Figure S14. Characteristic of *Hd1* haplotype.
(A) Bar plot for DTH in 2019, 2020, and 2021 for 252 overall ETN lines. *, ** shows p-values by two-sided student's t-test <0.05, 0.01, respectively. (B) Changes in haplotype composition in ETN lines with functional *Hd1* by the breeding period in Fukui. I, II, III, and IV: see Table1. *** above arrow indicates the significance at 0.1% level, in a Fisher's exact test that allows to reject the hypothesis that the ratio of haplotypes or polymorphisms in one period was maintained in the period pointed to by the arrow.

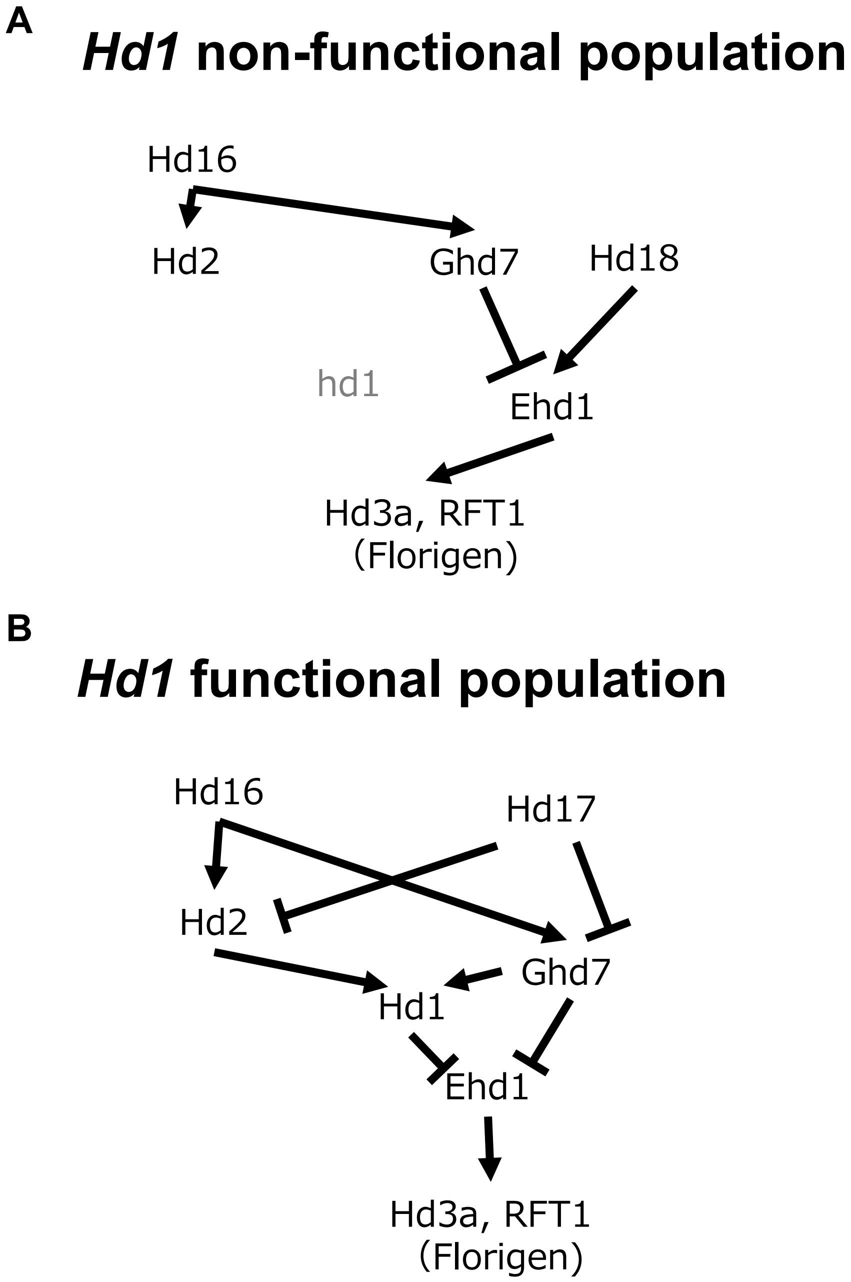

Figure S15. Gene Networks explaining *Hd16, Hd17,* and *Hd18* interactions.
(A) *Hd16* and *Hd18* interaction in the *Hd1* non-functional population. Under conditions of *Hd16* function (i.e., suppression of Ehd1 by Ghd7), *Hd18* competitively promotes flowering by enhancing *Ehd1* function, whereas, in the absence of *Hd16* function, *Ehd1* can function fully with or without *Hd18*.
(B) *Hd16* and *Hd17* interaction in the *Hd1* functional population. *Hd16* and *Hd17* competitively target Hd2 and Ghd7, where Hd16 activating function is more effective. In the Hd16-deficient background, the Hd17 function is not impaired and thus its function becomes more effective.

A

	Hd16/Hd17	*Hd1* Chr6 -	*Hd16* Chr3 33,002,789	*Hd17* Chr6 2,235,191	*Hd18* Chr8 2,388,554	*HESO1* Chr1 36,355,847	*OsCOL4* Chr2 23,990,288	*Hd6* Chr3 31,512,460	*Hd4* Chr7 9,154,394	*Hd2* Chr7 29,623,803	*GATA28* Chr11 4,433,209	Peak 1 Chr3 25,791,663	Peak 4 Chr6 22,504,254	Peak 5 Chr7 15,565,751
Population 1	L/E	HapA	0	0	0	0	0	0	0	0	0	0	0	0
	L/L	HapA	0	1	0	0	0	0	0	0	0	0	0	0
	E/E	HapA	1	0	0	0	0	0	0	0	0	0	0	0
	E/L	HapA	1	1	0	0	0	0	0	0	0	0	0	0
Population 2	L/E	HapA	0	0	1	0	0	0	0	0	0	0	0	0
	L/L	HapA	0	1	1	0	0	0	0	0	0	0	0	0
	E/E	HapA	1	0	1	0	0	0	0	0	0	0	0	0
	E/L	HapA	1	1	1	0	0	0	0	0	0	0	0	0
Population 3	L/E	HapB	0	0	0	0	0	0	0	0	0	0	0	0
	L/L	HapB	0	1	0	0	0	0	0	0	0	0	0	0
	E/E	HapB	1	0	0	0	0	0	0	0	0	0	0	0
	E/L	HapB	1	1	0	0	0	0	0	0	0	0	0	0

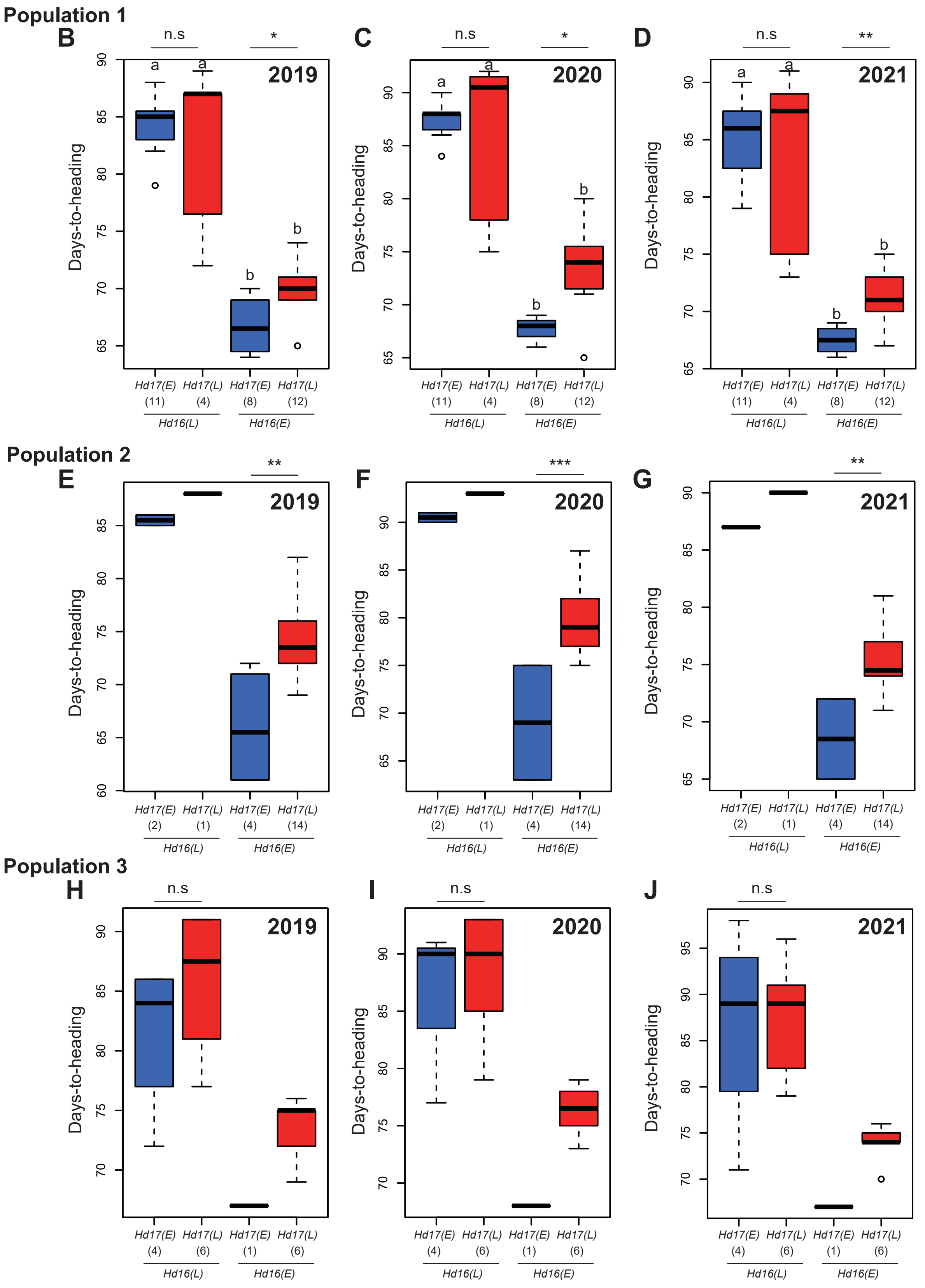

Figure S16. Gene by gene (GxG) interaction of *Hd16* and *Hd17* in the population with same genetic background.
(A) Combination of polymorphisms in known flowering genes and GWAS peaks. 0 indicates Reference, 1 indicates alternative popymorphism. (B-J) Boxplot for DTH in isogenic populations. (B-D) Population 1: Hap A of *Hd1* and *Hd18(E)*. (E-F) Population 2: Hap A of *Hd1* and *Hd18(L)*. (H-J) Population 3: Hap B of *Hd1* and *Hd18(E)*. Other known-flowering gene and the polymorphisms with the highest -$\log_{10}(P)$ in Peak 1, 2, 5 in overall 252 ETN population GWAS (Figure 2A) were also isogenic. *, **, *** shows p-values by two-sided student' s t-test <0.05, <0.01, <0.001, respectively. Different letters indicate significant differences with p-values <0.05 by Tukey-Kramer multiple comparison test. The number of lines in the ETN population is shown in parentheses.

A

Lines	*Hd1*	*Hd16*	*Hd18*	DTH_2014	DTH_2015_1	DTH_2015_2
NIL1	E	E	E	-7	-6	-5
NIL2	E	E	L	-3	-3	-3
NIL3	E	L	E	-3	-2	-3
NIL4	E	L	L	1	1	2
NIL5	L	E	E	11	8	10
NIL6	L	E	L	16	13	16
NIL7	L	L	E	22	22	25
NIL8	L	L	L	28	29	34

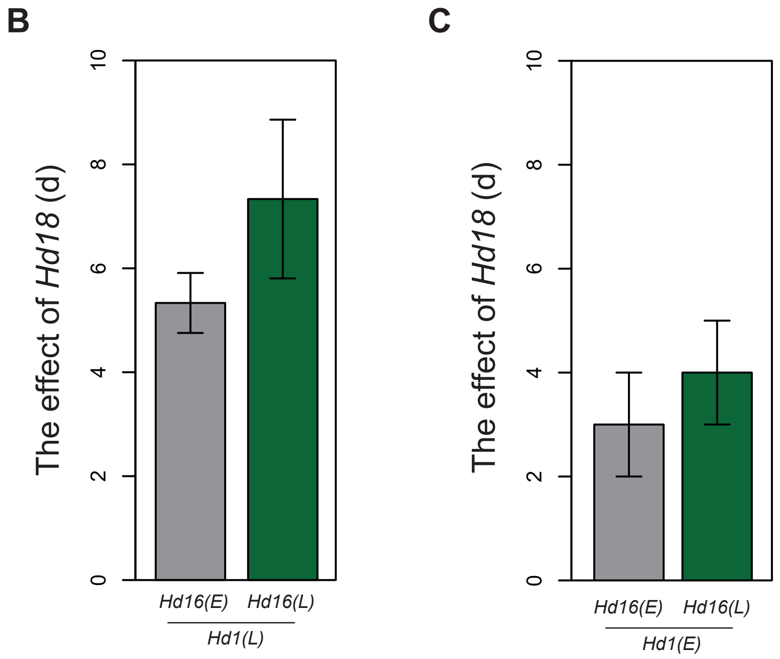

Figure S17. Gene by gene (GxG) interaction of *Hd16* and *Hd18* using NIL lines of Tohoku 206 and Koshihikari reported in Ishimori et al. (2020).
(A) Summary data reported in Ishimori et al. (2020). (B) Barplot for the effect of *Hd18* in *Hd16(E)* and *Hd16 (L)* backgrounds with *Hd1(L)* (B) and *Hd1(E)* (C). Barplot shows the average of DTH_2014, DTH_2015_1 and DTH_2015_2.

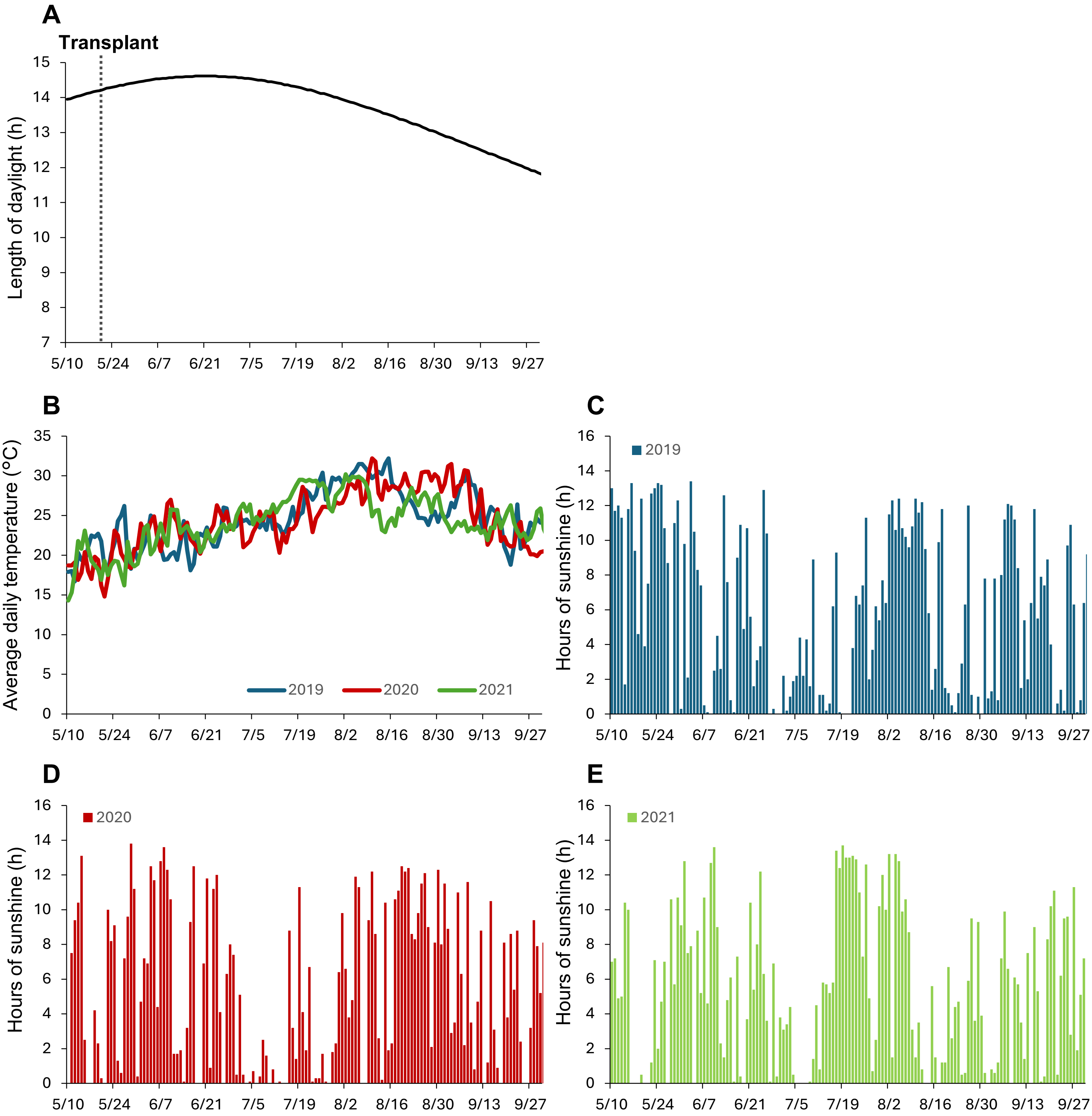

Figure S18. Environmental condition in Fukui.
(A) Length of daylight in Fukui (36.1°E, 136.2°N). (B) Average daily temperature in 2019, 2020, and 2021. (C) Hours of sunshine in 2019. (D) Hours of sunshine in 2020. (E) Hours of sunshine in 2021.
